# Randomization and allocation procedures for master protocol trials of single-arm studies

**DOI:** 10.1177/09622802261425426

**Published:** 2026-03-25

**Authors:** Peter Jacko, Günter Heimann, Tom Parke

**Affiliations:** 1 Department of Management Science, 4396 Lancaster University, Lancaster, UK; 2 Berry Consultants, Abingdon, UK; 3 Advanced Quantitative Sciences/Pharmacometrics, Novartis Pharma AG, Basel, Switzerland

**Keywords:** clinical trial, allocation procedure, randomization, master protocol, platform design, umbrella design, single-arm study, proof of concept, rare disease, simulation

## Abstract

In this paper we propose and examine randomization and allocation procedures in master protocol trials where each subtrial is performed as a single-arm study, which is motivated mainly by rare diseases. The subtrial analysis is done by comparing the single-arm observations data to historical data or expert judgment and following prespecified decision rules, without using data from any other study in the master protocol trial. The objective of this paper is to examine relevant options for participant allocation procedures in a variety of patterns in which the subtrials enter the master protocol trial, such as umbrella, platform and perpetual patterns. Our simulation study shows that our proposed allocation procedures using the predictive probability of current study success may bring substantial efficiency gains in the master protocol trial, making a clever allocation of the in-trial participants to achieve study success declarations sooner, benefiting the out-of-trial population.

## 1. Introduction

Master protocol trials, such as platform, umbrella and basket trials,^1,2^ are increasingly used for rare (a.k.a. orphan) diseases because they offer gains that may outweigh the methodological issues associated with them.^3,4^ The purpose of such trials is to provide a common infrastructure for sponsors to make it easy to investigate an intervention in a rare disease in an intervention-specific subtrial, defined in the corresponding intervention-specific appendix to the master protocol. The gains often cited for master protocol trials over a series of separate trials include (i) *statistical efficiencies* achieved from the same number of trial participants by methods taking advantage of features such as sharing of the control data, (ii) *operational efficiencies*, because trial design, site selection, staff training, database set-up and other infrastructure require fewer resources, and (iii) *health benefit* for trial participants, as the combination of suitably chosen subtrial-entry patterns, decision rules for early subtrial stopping, and allocation procedures leads to allocating more trial participants to better subtrials sooner. The three types of gains lead to a multiplicatively faster drug/intervention development and thus higher overall health benefit for the target population.^5^

The procedure for allocating participants when there are several subtrials open for enrollment is a key element of the design of a master protocol trial. This topic has attracted a lot of research attention. For master protocols that include one or more control arms, one of the main questions is the allocation to the control(s), which can either be one common control whose data are shared by all subtrials for their individual analyses, or an intervention-matched control in every subtrial, or both.^6,7^ Moreover, allocation between the subtrials can be made unequal or even adaptive, using early subtrial stopping or response-adaptive randomization, both of which can be specified in a number of ways depending on the trial objectives (e.g. participants benefit, statistical power, etc.), see Thall and Wathen,^8^ Wason and Trippa,^9^ Wathen and Thall,^10^ Viele et al.^11^ The choice of the allocation procedure heavily affects the operating characteristics of each subtrial.^12^ It may introduce or amplify a number of undesirable biases, which need to be properly investigated and evaluated by the trial designers, especially when the trial data is planned to be used for marketing authorization applications.^13^

Practically all the research in this area has focused on master protocol trials that include one or more controls (shared or intervention-specific). However, in a trial for a rare disease, one may not always be able to include a control, for example because there is no approved intervention on the market, because it may be unethical to use a placebo control, or because the patient population is very small.^14^ Single-arm (a.k.a. non-randomized) studies may become an acceptable, preferred, or even the only option in these situations. Bell and Tudur Smith^15^ reported that single-arm studies compose a relevant proportion of clinical trials, 29.6% for non-rare and 63.0% for rare diseases.

Master protocol trials consisting of single-arm subtrials have been proposed recently but, to the best of our knowledge, their performance has not yet been investigated. The EU-PEARL project published two master protocols: one for neurofibromatosis type 1 and one for neurofibromatosis type 2.^16,17^ These master protocols consist of single-arm subtrials for different manifestations of neurofibromatosis type 1 or 2. They allow for randomization between subtrials when several interventions are available at the same time.^18^ The European Medicines Agency^19^ reflection paper on single-arm trials also considers that these could be performed under a master protocol:

An example for such a trial would be a particular kind of platform trial where several investigational treatment arms are included but which are not formally compared, and which can be viewed as a series of single-arm trials.

These examples can be viewed as master protocols that consist of a number of single-arm studies, run in series and/or in parallel. An allocation procedure needs to be employed across subtrials if several of these are open for enrollment at the same time. The purpose of using a response-adaptive allocation procedure in such a trial is to skew the enrollment of participants to subtrial(s) that are most likely to declare success, which brings health benefit to both in-trial participants and the out-of-trial patient population. This purpose differs from conventional trials with a prespecified overall trial sample size, where response-adaptive allocation procedures aim to increase the group size for specific intervention(s). In a master protocol with prespecified subtrial sample sizes, all subtrials will eventually get their planned allocation of trial participants. Because of this, the statistical operating characteristics like statistical power and type I error (derived under the assumption of no temporal trends) for each subtrial are not affected by using a response-adaptive allocation procedure. The above argument is stated assuming a fixed sample size for each single-arm subtrial, but applies analogously also to more complex trials with early subtrial stopping.

In this paper, we examine allocation procedures in a master protocol trial where all subtrials are single-arm studies. The research was motivated by the EU-PEARL master protocols for neurofibromatosis. The allocation procedures explored here are based on the concepts of frequentist predictive probability^20^ and conditional probability of study success, which lend themselves easily for also defining response-adaptive allocation procedures. Our main objective is to explore the effects of response-adaptive allocation on the (non-statistical) operating characteristics of such a trial, more specifically, whether these procedures are capable of sooner completion of enrollment to the subtrials that are more likely to declare success at the final analysis. We present the results of an investigation of three allocation procedures over a number of different scenarios. The performance is evaluated using a newly developed and open-source clinical trial designer and simulator “Single-arm,”^21^ building on SIMPLE, an R software for simulating clinical trials.^22,23^

The paper is organized as follows. In Section 2 we describe procedures for allocating participants to subtrials that we consider relevant for a master protocol trial of single-arm studies, proposing a family of allocation procedures using predictive probability of current study success as our main contribution. In Section 3 we describe the setup of our simulation study, including the definitions of the different subtrial-entry patterns to be considered, the simulated sets of assumptions about unknown truths, and the master protocol trial design variants to be compared. In Section 4, simulation results comparing the performance of design variants across a comprehensive set of scenarios are presented. In Section 5 we describe three individual simulations that are illustrative of how the allocation procedures behave. We discuss the strengths and limitations of our methodology as well as potential further research topics in Section 6.

## 2. Randomization and allocation procedures

### 2.1. EU-PEARL master protocols

In this subsection, we briefly review the approach to randomization taken in the EU-PEARL master protocols for neurofibromatosis type 1 (NF1) and 2 (NF2), which serve as the motivation for this paper. Each of these protocols includes several manifestations of the disease. Patients diagnosed with one of these manifestations are eligible to be enrolled to the corresponding trial. Both the NF1 and NF2 trials have been set up as platform-basket trials (including several manifestations under one master protocol), because patients may switch between manifestations over time. Technically, they are a collection of manifestation-specific platform trials. These in turn consist of many single-arm subtrials, which are defined by the intervention being applied and the manifestation being treated. In other words, if an intervention is tested for two different manifestations, then this defines two subtrials. If there are two interventions tested for one manifestation, then this also defines two subtrials.

The inclusion and exclusion criteria as well as the primary endpoint differ between manifestations. Within a manifestation, the same endpoint is used across all subtrials and the inclusion and exclusion criteria at the master protocol level are also the same. However, there may be subtrial-specific inclusion and exclusion criteria that may be specified in the corresponding intervention-specific appendices to the master protocol. An example of a subtrial-specific exclusion criterion is that patients who have been treated with the intervention that is being tested in the subtrial before cannot be enrolled to the subtrial. Pretreatment with the intervention under investigation could have occurred outside of the NF1 or NF2 trials, or within, when treating a different manifestation (as patients can suffer from different manifestations of neurofibromatosis). A patient must meet the inclusion and exclusion criteria at the master protocol level and give consent to participate in the trial.

When there are several manifestation-specific subtrials that are open for enrollment at the same time, instead of being randomized directly to subtrials, participants are randomized to preference sequences in order to allow for non-eligibility. For each participant, the randomization procedure assigns an allocation preference sequence of the available subtrials. For example, if subtrials A , B , and C are enrolling, all their possible permutations are considered, for example B≻A≻C means that B is preferred over A which is in turn preferred over C , and one of them is assigned to the participant. The participant will be allocated to the first subtrial in this preference sequence for which they meet the subtrial-specific inclusion and exclusion criteria. This approach was chosen for operational simplicity, to avoid participant-specific randomization procedures (i.e. separate randomization procedures for participants who meet the subtrial-specific inclusion and exclusion criteria for A and B , and for participants who meet them for A and C , etc.). More details on the study design and the randomization approach can be found in Dhaenens et al.^18^

### 2.2. Proposed randomization and allocation procedures

In this subsection, we develop and describe a number of different procedures for allocating participants among subtrials at the master protocol level. The subsection is written without assuming a specific endpoint type, final analysis method or decision rule. More specific considerations are discussed in subsection 2.3.

The allocation procedures considered can be response-agnostic (ignoring all the accumulated endpoint observations) or response-adaptive, and for a given participant they can result in an allocation that is random or deterministic (conditional on available data). Note that if there is only one subtrial open for enrollment, all the allocation procedures result in a deterministic, unblinded allocation of the participant to that subtrial. In the rest of the paper, we consider that all the participants have the same covariate (like manifestation in the EU-PEARL trials). If there were several covariates, we could apply the allocation procedure adjusting it independently to each covariate.

In a master protocol for a rare disease, stakeholders might be interested in a trial objective of allocating participants quicker to those subtrials that are more likely to declare success, to make an efficient use of the rare patient population. Another objective could be to ensure fair or regular allocation of participants to subtrials that are open for enrollment. These different objectives are reflected in the participant allocation procedures that we evaluate in this paper.

To formally define different randomization and allocation procedures, we need to introduce some notation. Suppose that subtrials s=1,…,S ( S≤+∞ ) enter the master protocol trial over its duration, with a planned sample size of $N_{s}$ per subtrial. The intervention of subtrial s has an underlying true but unknown endpoint parameter denoted by $\theta_{s}$ .

When a newly enrolled participant t=1,2,… needs to be allocated, let $N_{s}(t)$ denote the number of allocated participants in subtrial s , and let $N(t)=N_{1}(t)+\cdots +N_{S}(t)$ denote the total number of enrolled participants to the master protocol trial. To accommodate delayed endpoint observations, suppose further that there are endpoint observations of $n_{s}(t)\leq N_{s}(t)$ participants available in each subtrial s , and let $n(t)=n_{1}(t)+\cdots +n_{S}(t)$ denote the total number of endpoint observations across all subtrials at the time of enrollment of participant t .

In order to allocate participant t , we consider only the subtrials that are currently open for enrollment, and denote by S(t) their indices, and by S(t) their number, assuming S(t)<+∞ for every t . With the $n_{s}(t)$ observations in subtrial s , given the observed endpoint cumulative sum $Y_{s}(t)$ by the time of enrollment of participant t , one can calculate the conditional probability $p_{s}(t)$ or the predictive probability $\pi_{s}(t)$ of declaring success at the end of the subtrial. We further use $\lambda_{s}(t)$ to indicate the probability with which participant t is randomized to subtrial s∈S(t) . We will use a superscript to indicate an allocation procedure when $p_{s}(t)$ , $\pi_{s}(t)$ and $\lambda_{s}(t)$ are procedure-specific.

#### 2.2.1. Equal fixed randomization

The simplest allocation procedure is to just randomize participant t equally among the available subtrials, *equal fixed randomization* ( *EFR* ), that is,

(1) $$\lambda_{s}^{EFR}(t)=\frac{1}{S(t)}$$

for all subtrials s∈S(t) . With this procedure, no subtrial is favored over the other from the perspective of participant t . However, it results in giving the advantage of sooner expected completion to subtrials that have already got more allocated participants.

#### 2.2.2. Oracle

The next allocation procedure is based on the conditional probability (the conditional power)

(2) $$p_{s}(t):=P_{\theta_{s}}\{\text{subtrial }s\text{ will declare study success}|Y_{s}(t)\}$$

that the subtrial will declare success at the final analysis given the observed endpoint cumulative sum $Y_{s}(t)$ by the time of enrollment of participant t . If there are no observations available for intervention s , then the conditional probability (2) reduces to the unconditional probability (the power) $P_{\theta_{s}}\{\text{subtrial }s\text{ will declare study success}\}$ .

This allocation procedure, which we call *Oracle* ( *Ora* ), allocates the participant t deterministically to the subtrial with the highest conditional probability (the *prevailing* subtrial). If there are two or more prevailing subtrials, the participant is randomly allocated to one of these with equal probability. Obviously, this procedure cannot be applied in practice, since the true endpoint parameters $\theta_{s}$ are unknown. In a simulation study, however, the endpoint parameters can be assumed, and this allocation procedure can serve as a reference, making allocations of participants to subtrials based on correct probabilistic predictions about the subtrials’ chance to declare success.

Note that this procedure will typically allocate more participants than *EFR* in a deterministic way, either because the data of different subtrials will often not be identical (and therefore there will not be a tie) or because it will more frequently have only one subtrial open for enrollment (because some subtrials will complete quicker). We will illustrate its behavior in Section 5.

#### 2.2.3. Predictive probability

The third allocation procedure, *Predictive Probability* ( *PrP* ), replaces the conditional power used to define the Oracle procedure by the predictive probability $\pi_{s}(t)$ of declaring success at the end of the subtrial, given the observed endpoint cumulative sum $Y_{s}(t)$ . Analogously to *Ora* , participant t is allocated deterministically to the subtrial with the highest predictive probability (the prevailing subtrial), and if there are several prevailing subtrials, the participant is randomly allocated to one of them with equal probability.

These predictive probabilities can be calculated based only on observed endpoint data, with no unknown endpoint parameters needed. When there are no observed endpoint data available in subtrial s (i.e. when we do not have a well-defined estimate of $\theta_{s}$ ), the predictive probability will be artificially set to 1 , reflecting the expectation that a new subtrial should be likely to declare success and should get a chance to start collecting endpoint observations regardless the joint state of the trial.

Such a predictive probability has to be obtained based on the analysis described in the master protocol or in the intervention-specific appendix for the subtrial. It depends upon the type of endpoint, the primary analysis method and the decision rules. We discuss this in more detail and describe the calculation of PrP in subsection 2.3, after introducing the randomized PrP procedure which also employs PrP values.

#### 2.2.4. Randomized predictive probability

Both these procedures ( *Ora* and *PrP* ) depend on observed endpoint data and often result in deterministic allocation (unless there is more than one prevailing subtrial). These procedures are designed to favor the subtrial(s) most likely to declare success at the final analysis. However, if the first participants allocated to a subtrial happen to accumulate low-valued endpoint observations, then the probability of declaring success at the final analysis can become and will remain small, so further participants will predominantly be allocated to the other subtrials. This feature may be undesirable especially when such random low-valued observations happen in a better subtrial.

In order to avoid this, we propose randomized procedures defined as a mixture of the *PrP* and the *EFR* procedures. For a given fraction 0≤ε≤1 , we define the *Randomized Predictive Probability* procedure, denoted by ε - *PrP* . For every participant t , ε - *PrP* uses a Bernoulli random variable $U_{t}$ with $P\{U_{t}=1\}=\varepsilon$ to decide whether the *PrP* procedure ( $U_{t}=0$ ) or the *EFR* procedure ( $U_{t}=1$ ) is used for the allocation of this participant. More precisely, if, for participant t , there are S(t) subtrials available and $S^{*}(t)$ (indexed by $S^{*}(t)$ ) prevailing subtrials according to *PrP* , the participant is allocated to subtrial $s\notin S^{*}(t)$ with probability

(3) $$\lambda_{s}^{\varepsilon -PrP}(t)=\frac{\varepsilon }{S(t)}$$

and to subtrial $s\in S^{*}(t)$ (a prevailing subtrial) with probability

(4) $$\lambda_{s}^{\varepsilon -PrP}(t)=\frac{\varepsilon }{S(t)}+\frac{1-\varepsilon }{S^{*}(t)}.$$

That is, in case of several prevailing subtrials, the procedure uses equal randomization among them. This procedure is analogous to the ε -greedy procedure well-known in reinforcement learning (see, e.g. Kuleshov et al.,^24^), with *PrP* being the particular greedy procedure. When ε can be easily written as a percentage, we will use an alternative nomenclature *ε⋅100% -PrP* , for example 40% - *PrP* when ε=0.4 . Note that *PrP* is recovered as 0% - *PrP* and *EFR* is recovered as 100% - *PrP* .

### 2.3. Discussion

The predictive probabilities that are required for the *PrP* and *ε -PrP* allocation procedures can always be defined (in the Bayesian manner as long as it is possible to specify a prior distribution, while in the frequentist manner as long as there is at least one endpoint observation). How to obtain them depends upon the endpoint, analysis and decision rules that are used in each single-arm subtrial. An example where this was done in a frequentist manner for binary endpoint is provided in Heimann et al.,^20^ which we summarize in Appendix A.2 and where provide the formula for calculating the frequentist predictive probability that we use in our simulation study for *PrP* and *ε -PrP* .

Similarly, in Appendix A.3 we provide the formula for calculating the conditional probability for binary endpoint that we use in our simulation study for *Ora* . While we have defined *Ora* using the unknown true endpoint parameter $\theta_{s}$ in order to provide a benchmark, analogous calculation could be done in practice using an assumed or estimated parameter instead of $\theta_{s}$ like it is conventionally done, for instance, in conditional power calculations for adaptive designs with unblinded sample-size re-assessment. However, we believe that *PrP* does the prediction better than plugging-in a point estimate or an assumed value of the parameter because it captures the uncertainty in the observed data, while the plug-in method does not. This is based on our own observations in simulations of such a procedure (not reported in the paper). A related discussion is also given in Section 3.2 and Appendix A.2 of Heimann et al.^20^.

For our simulation study, all subtrials use the same endpoint, the same analysis and the same decision rules, motivated by the EU-PEARL NF trials. For more details see Heimann et al.^25^ An advantage of using predictive probability as proposed here is that one can in principle apply the approach to master protocol trials when the decision rules, analysis, or even the endpoints differ across the subtrials. The common scale is the probability scale, and the common measure is the predictive probability to declare success at the end of the study given the data observed so far. How this predictive probability is calculated does not matter when defining the allocation procedures.

Both *Ora* and *PrP* procedures prioritize subtrials that are more likely to declare success, but they do not consider when such a subtrial success would happen or how many participants have been allocated but not yet observed due to endpoint observation delay. Also, they evaluate each subtrial separately, and thus ignore the joint state of the master protocol trial such as the number of subtrials open for enrollment or the existence of new subtrials waiting in a queue to join the trial as the currently open subtrials complete. These limitations may impact their performance especially if there are non-negligible differences between subtrials. For instance, *PrP* may not behave as expected if the observation delay for the endpoints differs materially. Since the predictive probabilities are set to 1 until there are endpoint observation data available, the *PrP* procedure will favor the subtrial with the longer observation delay for a longer time as a result of this convention, even if the corresponding intervention has lower response rate and/or more demanding decision rules, both of which lead to a lower chance to declare success. In our simulation study we assume that the same endpoint observation delay is used in all the subtrials. If the observation delays differ, one should conduct careful simulations to understand the (non-statistical) operating characteristics of our proposed procedures, or to modify them to avoid any undesired behavior.

The allocation procedures we have proposed are based on the predictive probability for a subtrial to declare success at the final analysis. They are not based on predicting the unknown endpoint parameter (e.g. response rate) of the intervention in the subtrials. These two concepts lead to the same ordering of subtrials if subtrial definitions (sample size, endpoint characteristics, analysis and decision rules), number of allocations and number of observations are identical, but they may disagree in general. That the allocation procedure favors the subtrial that is more likely to declare success is desirable from the sponsors’ point of view, who may have defined the decision rules to declare success following different objectives or research questions, answering of which is the main objective of the master protocol trial. In certain situations, other reasons, such as the change in the standard of care or change in the disease nature, may lead to some subtrials being stopped on ethical or scientific grounds for being obsolete and/or possibly harmful despite having a relatively high predictive probability.

Strictly speaking, the procedures defined in the previous paragraphs can only be applied when the inclusion and exclusion criteria of all subtrials are the same, so that every participant is eligible for every subtrial. This may not be the case, as discussed in subsection 2.1. This issue can be resolved by randomizing to allocation preference sequences of available interventions, as in the EU-PEARL NF trials. The *EFR* procedure can be modified to allocate participants to the allocation preference sequences of available interventions with probability 1/S(t)! , which results from completely random ordering of the S(t) available subtrials, as there are S(t)! possible preference sequences. The *Ora* , *PrP* and *ε -PrP* procedures can be adapted analogously, to allocate each participant to an allocation preference sequence with the interventions in the order of the conditional power or predictive probability, respectively, while creating a completely random ordering of the tied subtrials. This approach leads to a single randomization procedure regardless of differences in the inclusion and exclusion criteria of subtrials. Note, however, that the allocation probabilities to the subtrials would depend upon the individual participants if there were subtrial subpopulations. Our approach can be employed, but investigation of such a situation is beyond the scope of this paper. For our simulation study, we have assumed that all participants are eligible for all subtrials.

A reader familiar with Bayesian response-adaptive randomization (BRAR) (cf. Thall and Wathen^8^) might wonder whether *Ora* and *PrP* could have been defined by randomizing to each subtrial s proportionally to $p_{s}(t)$ and $\pi_{s}(t)$ , respectively, instead of deterministically allocating to the subtrial(s) with their highest value(s). Unfortunately, both such versions would be ill-defined and hard to interpret. Proportional randomization is used in BRAR procedures, in which $\pi_{s}(t)$ represents the posterior probability of an intervention having the highest mean, that is the data from all the interventions is considered when calculating these. Therefore, such values add up to 1 when summed up over all the interventions, defining valid probabilistic weights for randomization. Due to Bayesian priors, the limiting values of 0 and 1 are not possible, and even after using additional BRAR tweaks such as exponentiating or clipping of small values to zero and subsequent re-normalization, these remain mathematically valid and continue providing a reasonable interpretation. In the reinforcement learning literature, there are proofs that using such proportional randomization leads to asymptotically (as the trial size grows to infinity) optimizing the objective of the health benefit for trial participants (see, e.g. Russo et al.^26^). Contrary to BRAR, the values in *PrP* and *Ora* are predictive and conditional probabilities, respectively, calculated for each subtrial in isolation. They can assume any value between 0 and 1 , including the limiting values. They do not add up to 1 naturally, and the interpretation of the number obtained by adding them up is the expected number of success declaration by the end of the master protocol trial. We do not see any natural interpretation of a randomization defined proportionally to those values, nor what objective this could optimize. Furthermore, normalization by the sum of these values would not be mathematically valid; for instance, when all π ’s are zero (which happens often, as long as there is no response observed or when the number of observed responses is too small shortly before reaching completion), the denominator is an undefined division ( 0/0 ). For that situation, we could artificially define to use *EFR* instead; actually, this is what both *PrP* and ε - *PrP* do in that situation. In another frequent situation, when one subtrial’s $\pi_{s}$ is positive and all the others are zero, proportional randomization would allocate the subtrial with positive $\pi_{s}$ deterministically; actually, this is exactly what *PrP* does, while ε - *PrP* uses *EFR* with probability ε , thus enforcing randomization. Similarly, when there are several subtrials with positive $\pi_{s}$ , proportional randomization would randomize between these; *PrP* would prioritize the subtrial with highest $\pi_{s}$ , while ε - *PrP* would ensure some level of randomization to each subtrial (including those with zero $\pi_{s}$ ). Based on the above, we believe that the randomized procedure as defined above, ε - *PrP* , is the appropriate one, if and when randomization is required.

## 3. Simulation study

We have designed a simulation study which mimics the EU-PEARL trial for a rare disease such as neurofibromatosis described in Dhaenens et al.^27^ or Heimann et al.^20^ In the following subsections we provide more detail on the simulation study set-up.

### 3.1. Enrollment of trial participants

We consider a discrete-time simulation model, that is regularly-spaced time periods, where each period would—in the context of a rare disease—typically be interpreted as a month or as a quarter. The enrollment of participants to the master protocol trial takes place with one enrolled participant per period, so participant t=1,2,… is enrolled at the beginning of period t , and thus the number of previously enrolled participants N(t)=t−1 .

### 3.2. Subtrial-entry patterns

One of the key features of master protocol trials is that they can prespecify that new subtrials can enter during the course of the trial as new interventions become available for investigation. Sometimes, there are several interventions available when the trial starts, sometimes the trial may be set up without an intervention and the timing when interventions become available is unknown. The effect of different timings when subtrials enter master protocol trials is not well understood.^28,29^ When there already are several subtrials open for enrollment, it is a strategic decision whether to allow additional subtrials to enter, which ones to allow, and when to let them enter the trial. Such a decision will impact the trial participants, the speed of completion of the currently enrolling subtrials, the attractiveness of the trial to potential future participants and the attractiveness of the trial to the sponsors of potential future interventions.

The simulation results are presented in three subtrial-entry patterns:

- the *umbrella* pattern, which has a fixed number of subtrials, all entering from the beginning of the trial and concurrently open for enrollment (until completing their respective sample sizes), sometimes also called a multi-arm trial;
- the *platform* pattern, which has a fixed number of subtrials entering the trial over time in a staggered fashion, leading to a varying number of them concurrently open for enrollment;
- and the *perpetual* pattern, which is a variant of the platform pattern with an unbounded stream of subtrials entering the trial over time, but with a fixed limit on the number of subtrials concurrently open for enrollment.

These patterns are illustrated in Figure 1, where the darkest color highlights the time of entry into the master protocol trial, and fading illustrates the uncertainty of the enrollment completion time of a subtrial. In our simulation study, in the platform and perpetual pattern we assume that there is 1 subtrial from the period 1 available for participant t=1 and a new subtrial becomes available every 12 periods. The illustrated entry times (in periods 49,61,… ) in the perpetual pattern are the earliest that a new subtrial would be able to enter the master protocol trial, as it needs to wait in a queue until one of the currently open subtrials completes enrollment if the limit on the number of concurrently open subtrials is reached.

**Figure 1.**
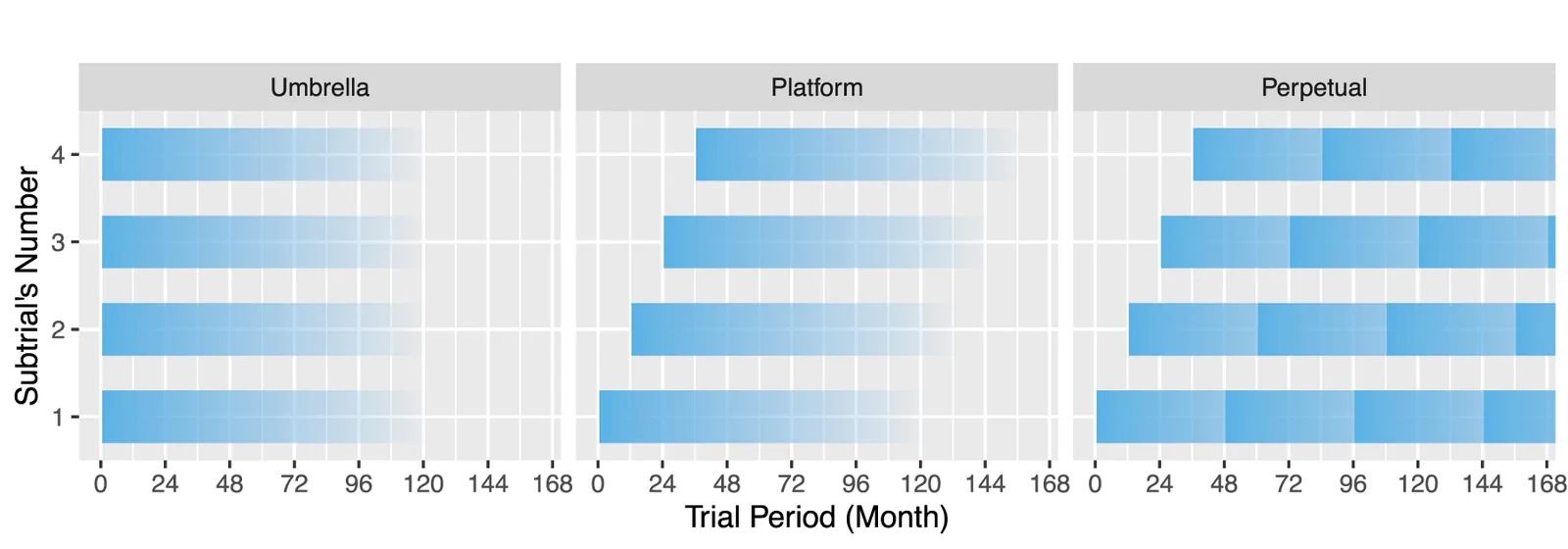
An illustration of subtrial-entry patterns.

In our simulation study we limited the number of open subtrials to four, as in Figure 1. For master protocols with umbrella or platform subtrial-entry patterns, a subtrial that completes enrollment is not replaced. In the perpetual pattern, the number of open subtrials is limited to four, and once this number is reached, a new subtrial (once it is available) can only enter when another one completes enrollment.

All three patterns may be applicable in different situations and are of interest on their own, often without being able to choose between them. The EU-PEARL master protocols discussed in subsection 2.1 fall into the perpetual pattern, with subtrials gradually entering whenever they become available. The total number of subtrials nor the limit on the number of concurrently open subtrials are not fixed in the protocol.

### 3.3. Subtrial endpoint

For each subtrial s , we consider binary (response / non-response) primary endpoint, with $\theta_{s}$ interpreted as the *response rate*; there are no secondary endpoints. The endpoint for an individual participant is observed 4 periods after enrollment, that is 4 months if the period is a month or 1 year if the period is a quarter. We assume that there are no early observations at earlier visits. Formally, this means that the endpoint of a participant t is not available when enrollment of the next four participants ( t+1,t+2,t+3,t+4 ) takes place. For the sake of focusing on key features of the proposed approach, we assume that there are no missing observations and no differences in the subtrial populations.

### 3.4. Response rate vectors

In the simulation scenarios presented here, we consider S=4 subtrials in the umbrella and platform patterns. For each subtrial s , the response rate of its intervention is $\theta_{s}\in \{0.1,0.2,0.3,0.4\}$ , which we call the type 1,2,3,4 , respectively. We denote, for example, the vector $\left(\theta_{1},\theta_{2},\theta_{3},\theta_{4})=(0.3,0.1,0.2,0.3\right)$ of subtrial response rates by its type sequence 3123. In the perpetual pattern, we limit the number of subtrials concurrently open for enrollment to 4 and for the subsequently entering subtrials we recycle the response rates of the first 4 subtrials. For instance, response rates vector 3123 corresponds to $\left(\theta_{1},\theta_{2},\theta_{3},\theta_{4},\theta_{5},\theta_{6},\theta_{7},\theta_{8},\theta_{9},\ldots )=(0.3,0.1,0.2,0.3,0.3,0.1,0.2,0.3,0.3,\ldots \right)$ . We have considered a comprehensive list of 18 different response rates vectors chosen to cover a range of different plausible real-life situations, but due to space restrictions we present here only 4 of them (1111, 1131, 1123, 3123), which are qualitatively representative of the whole list.

### 3.5. Subtrial analysis and decision rules

For each subtrial with its prespecified sample size, the decision of success declaration is taken as a result of the final analysis, which is performed at the end of the period during which the endpoint of the last enrolled participant of the subtrial is observed. There are no decisions taken at interim sample sizes.

The final analysis for each subtrial is as defined in the EU-PEARL master protocols^20,25^ based on confidence distributions, see Appendix A.1 for details. To define a decision rule for our simulation study, we chose the ineffective response rate to be $p_{0}=0.1$ , the desired response rate to be $p_{1}=0.2$ , and we set α=0.1 and β=0.5 . This final analysis method results in simplifying the decision rule into defining a cut-off value $c_{N}$ for sample size N so that a study success is declared in the final analysis if there are at least $c_{N}$ observed responses. Such a decision rule could be obtained from many other final analysis methods, so the use of the confidence distributions method is just an illustrative example rather than a necessary assumption. The statistical operating characteristics of single-arm studies using these decision rules are stated in Table 1 for a cross-product of considered response rates against pairs of sample sizes and cut-off values.

**Table 1.** Unconditional probability of study success declaration for different response rates $\theta_{s}\in \{0.1,0.2,0.3,0.4\}$ against pairs of sample sizes and cut-off values of a given single-arm study.

Sample size $N_{s}$	Cut-off $c_{N_{s}}$	0.1	0.2	0.3	0.4
17	4	0.08264062	0.45112380	0.79809299	0.95357707
24	5	0.08507489	0.54012267	0.88892478	0.98655089
40	9	0.01549531	0.40687287	0.88899082	0.99393537

The decision rules regard an intervention of subtrial s with a response rate $\theta_{s}$ smaller than or equal to $p_{0}$ as medically non-efficacious, or not of commercial interest. For our simulation study this means that all the response rate vectors containing 1 (all of the presented ones), contain at least one intervention (the one which is denoted as 1 ) that would not be regarded as of interest. We thus call any subtrial of type 1 a *null* subtrial and 1111 the *global null* response rate vector. To simplify the discussion below, we further call every non-null subtrial a *good* subtrial, and the subtrial(s) with the highest type out of those listed in a response rate vector the *best* subtrial(s). For instance, in 3123, subtrials s=1,3,4 are good, and subtrials s=1,4 are best. More generally, if different subtrials had different decision rules, a good subtrial could be defined as a subtrial whose unconditional probability to declare success at the final analysis is sufficiently high, for example higher than 50% ; and that unconditional probability could create an ordering to say which subtrial is *better* than another, but note that this would not necessarily match the ordering of the subtrial response rates.

### 3.6. Simulation scenarios

A simulation scenario is defined jointly by the *set of assumptions* about unknown truths and by a master protocol trial *design variant*. The set of assumptions that were varied includes the subtrial-entry pattern and the vector of subtrial response rates, defined in the previous subsections. We did not vary the assumptions about the participants’ enrollment.

The design variant includes the choice of sample size $N_{s}\in \{17,24,40\}$ for each subtrial s (with all subtrials in one simulation scenario having the same sample size, which we henceforth denote briefly by N ), and the allocation procedure ( *Ora* , *PrP* , *40% -PrP* , *70% -PrP* , *EFR* ). We did not vary the endpoint characteristics nor the decision rules.

Overall, we present results of 12 assumption sets, determined by 3 subtrial entry patterns and 4 response rate vectors, and we consider 12 design variants, determined by the 3 sample sizes and the 4 allocation procedures for each sample size, yielding 144 simulation scenarios altogether.

It is important to realize that the ordering of the subtrials’ response rates does not matter in the umbrella pattern regardless of the allocation procedure, nor does it matter when the allocation procedure is *EFR* , but it does matter in the platform and perpetual patterns when an adaptive allocation procedure is used. For instance, 3123 in the platform patterns means that subtrial 1 has a higher response rate ( $\theta_{1}=0.3$ ) than subtrial 2 ( $\theta_{2}=0.1$ ) which enters later. Therefore, adaptive procedures would, after seeing some observations, be expected to prioritize subtrial 1 to complete much sooner than subtrial 2 . If the response rates were the other way around, adaptive procedures would tend to prioritize subtrial 2 and therefore enrollment of participants to subtrial 1 would slow down. For the perpetual pattern this could mean that some subtrials remain in the trial with very slow enrollment or, in some extreme circumstances, could never complete. Such a situation can arise if the subtrials that enter later frequently are better than those that entered earlier.

## 4. Simulation results

The simulation results presented in this section are averages obtained from 50,000 individual simulation runs of each simulation scenario. Taking the averages means that we are reporting *expected* values of the metrics that are obtained via Monte Carlo simulation.

### 4.1. Number of correct success declarations

The figures in this section present the expected cumulative number of *correct* success declarations (vertical axis) as a function of time (horizontal axis) for the different response rate vectors (rows) and sample sizes (columns). There is a separate figure for each subtrial entry pattern (umbrella, platform, and perpetual). The different colors in these figures represent the different allocation procedures: *Ora* , *PrP* , *40% -PrP* , *70% -PrP* , and *EFR* .

A correct success declaration is a success declaration for a subtrial with an underlying response rate $\theta_{s}\geq 0.2$ . In the first row of the three figures shown in this section, all success declarations are therefore wrong, since all subtrials have the same true underlying response rate 0.1 . In the second row, only the success declarations for subtrial s=3 are correct, in the third row, only those for s∈{3,4} , and in the last row only those for s∈{1,3,4} are correct.

The master protocol trials with umbrella (called *umbrella trial* in what follows) and platform (called *platform trial* in what follows) subtrial-entry pattern include exactly four subtrials in our simulations. These master protocol trials end after 72 periods if the sample size per subtrial is N=17 (first column), 96 periods for N=24 (second column), and 164 periods for N=40 (third column), even in case of the platform trial as a new subtrial enters the trial every 12 periods, which is less than the subtrial sample size. The horizontal axis extends over 164 periods in all three columns, for better comparability. The expected cumulative number of correct success declarations is reached at the latest after 72 or 96 periods when N=17 or N=24 , respectively. This number is carried forward until the end of the horizontal axis (period 164 ), and appears as a plateau in the corresponding figures for the umbrella and for the platform trials.

For the umbrella and platform trials, the overall expected cumulative number of correct success declarations at the end of the trial (i.e. the plateau) is predetermined by the scenario, and can be obtained from Table 1. This table provides the probability to declare success for a subtrial as a function of sample size (rows) and true response rate (columns). Therefore, in an example when just one subtrial has a response rate $\theta_{s}=0.3$ and the rest have response rate $\theta_{s}=0.1$ (as in the second row of Figures 2 or 3), the overall expected cumulative number of correct success declarations equals this number ( 0.79809299 for N=17 , 0.88892478 for N=24 , and 0.88899082 for N=40 ). In the scenario 1123 where two subtrials have a true response rate $\theta_{s}\geq 0.2$ (one of 0.2 and one of 0.3 ) (as in the third row of Figures 2 or 3), the overall expected cumulative number of correct success declarations equals the sum of the corresponding numbers ( 0.45112380+0.79809299 for N=17 , 0.54012267+0.88892478 for N=24 , and 0.40687287+0.88899082 for N=40 ). For the scenario in the first row, where all true underlying response rates equal $\theta_{s}=0.1$ , no success declaration is correct. Therefore, the overall expected cumulative number of correct success declarations equals zero here.

**Figure 2.**
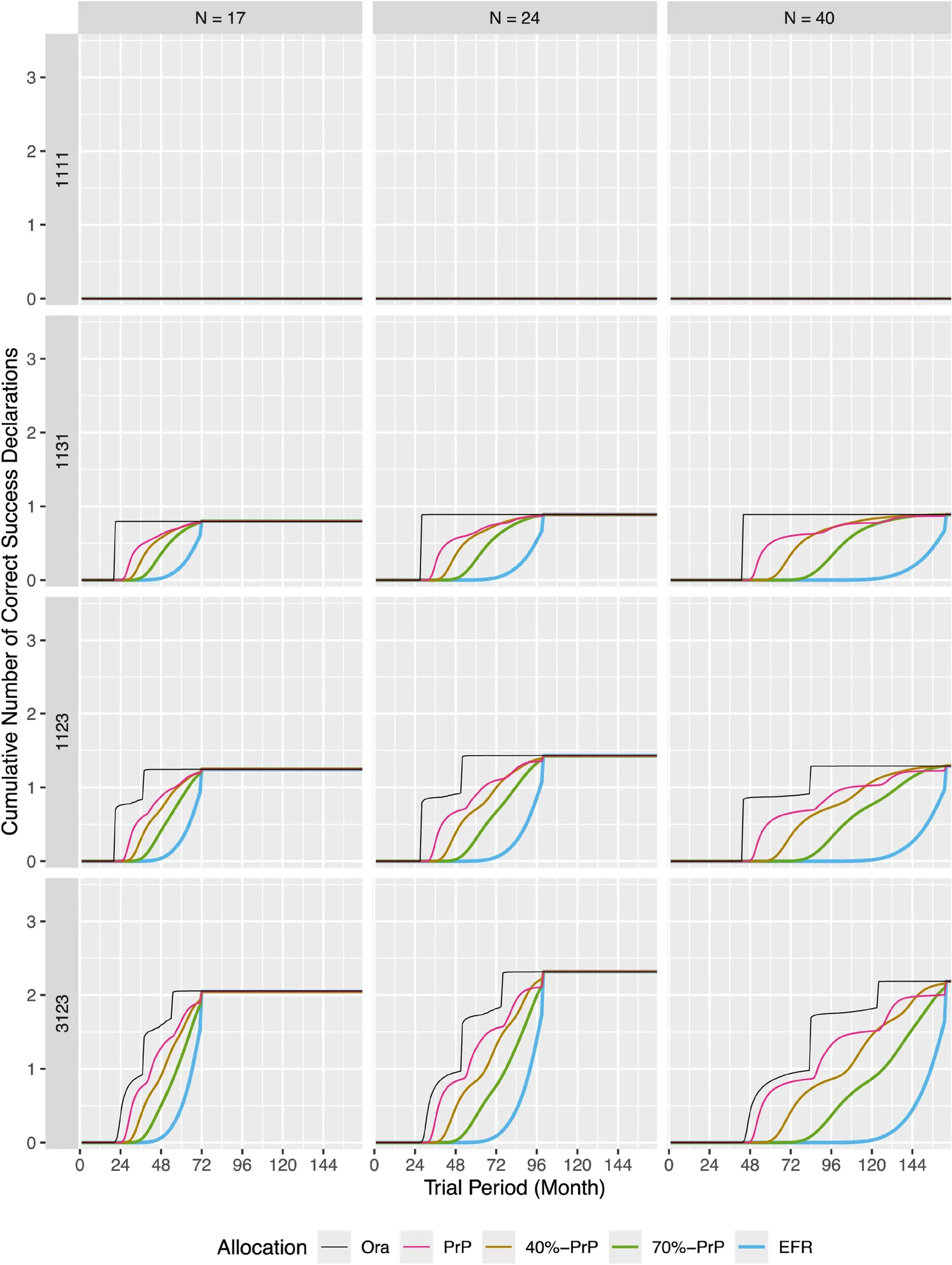
The cumulative number of correct success declarations in an umbrella trial.

**Figure 3.**
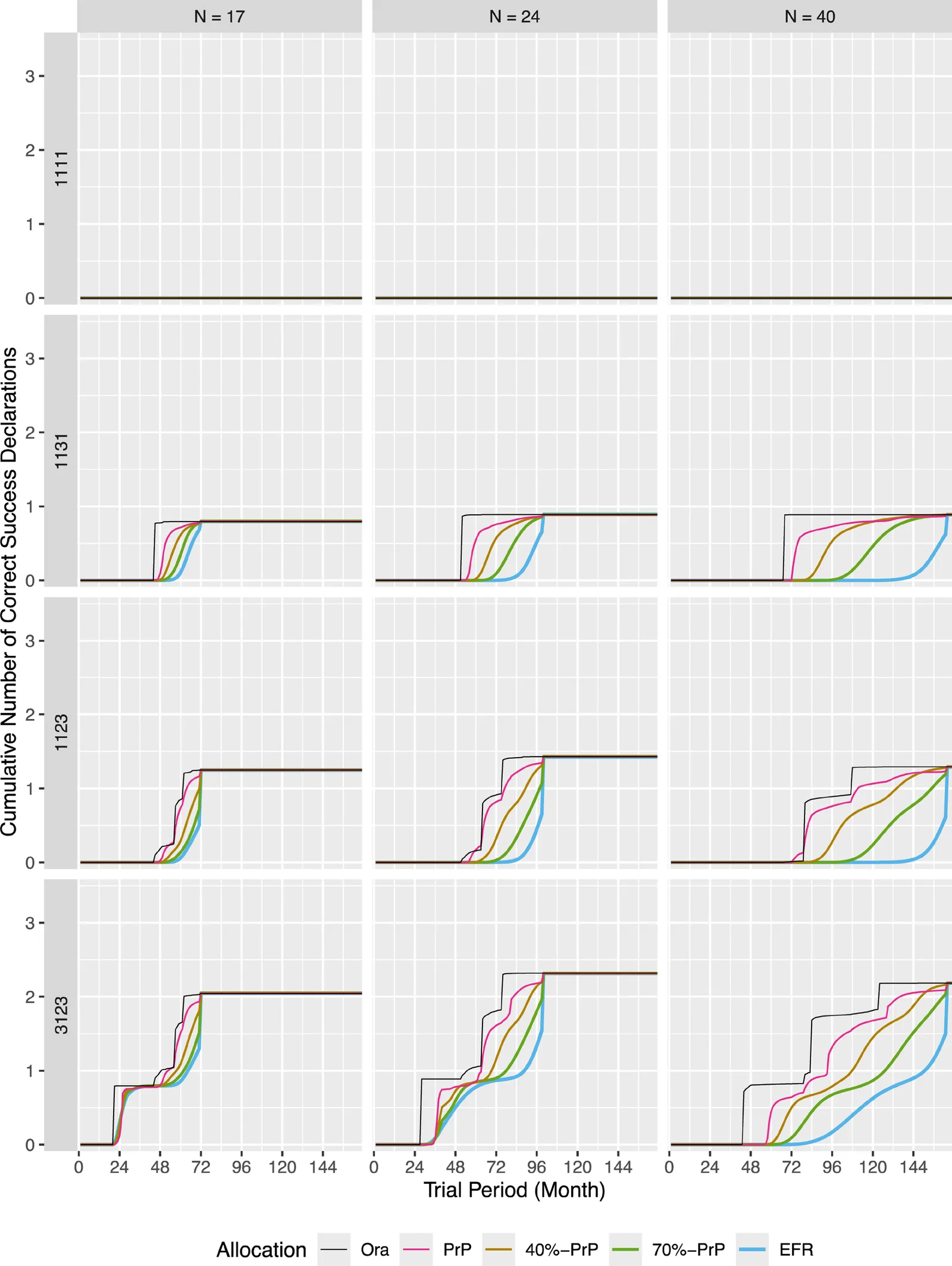
The cumulative number of correct success declarations in a platform trial.

For master protocol trials with a perpetual subtrial-entry pattern (called *perpetual trial*), the number of subtrials is not limited to four. Rather, there will always be maximum of four subtrials that are open for enrollment for any participant t . Therefore, the cumulative number of correct success declarations at period 164 cannot be calculated exactly, unlike for the umbrella and the platform trials. There is also no plateau for when N=17 or N=24 , see Figure 4.

**Figure 4.**
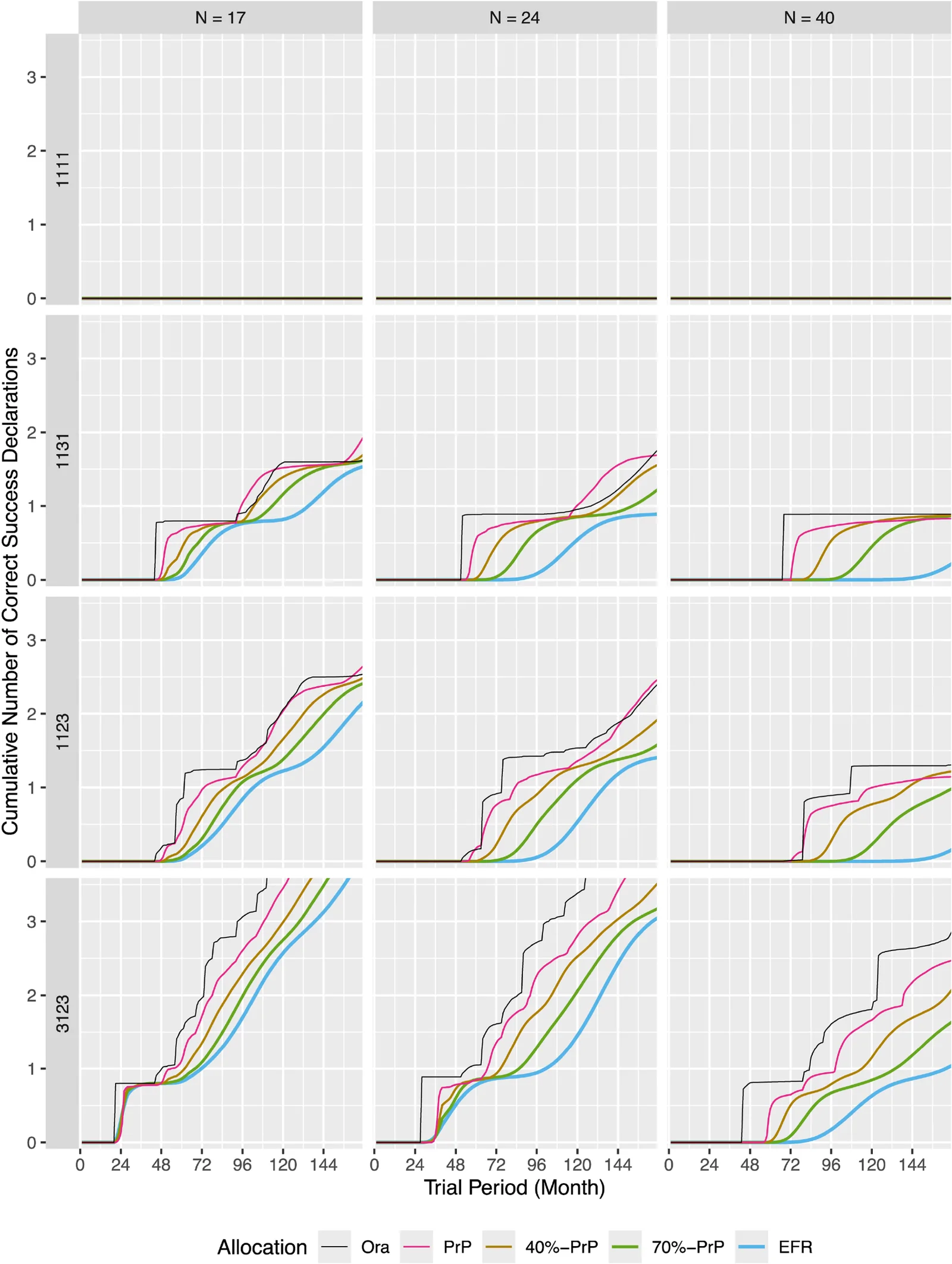
The cumulative number of correct success declarations in a perpetual trial.

#### 4.1.1. Umbrella trials

Figure 2 summarizes the results for umbrella trials. Since the overall expected cumulative number of correct success declarations is predetermined and does not require simulations, the information displayed lies in the time course, which describes the trajectory how this number is reached.

Not surprisingly, the slowest procedure is *EFR* (blue). Success declarations start towards the end of the trial, when the first of the subtrials accumulates all the observations. Since the *EFR* procedure allocates equally to subtrials irrespectively of how likely they are to declare success, the expected cumulative number of correct success declarations remains at zero or very close to it until the final third of the duration of the trial.

In contrast, the *Ora* procedure (black) tends to allocate participants to the subtrial with the highest response rate, which is the most likely to declare success given that all the decision rules are identical. In a scenario where three subtrials are null, and only one is good, the procedure allocates new participants to this one good subtrial first, and continues to allocate to it until it is complete. (There may be rare occasions where the initial data is unusually poor, though.) Therefore, the overall expected cumulative number of correct success declarations is achieved shortly after the minimal time needed for this subtrial to complete enrollment and to have all observations available. For instance, our simulations results of scenario 1131 say that this is on average (rounded to 4 decimals and subject to simulation error) in period 21.0026 for N=17 , in period 28.0035 for N=24 , and in period 44.0002 for N=40 , which (recalling the 4 -period observation delay) suggests that the chance of allocating to a non-best subtrial is negligible. This can be visually appreciated in Figure 9 in Appendix B, which is the equivalent of Figure 2 showing the probability mass instead of the cumulative distribution. Allocation to the other three subtrials occurs when the best subtrial has completed enrollment, but no further correct success declarations will occur, since these three subtrials are null ( $\theta_{s}=0.1$ ).

In the scenario 1123 (third row), the *Ora* procedure tends to allocate new participants to subtrial 4 first ( $\theta_{4}=0.3$ ). Once enrollment for this subtrial has completed, enrollment will switch to subtrial 3 ( $\theta_{3}=0.2$ ), and when this subtrial has completed enrollment, participants will be allocated to remaining null subtrials. The correct success declarations occur in two steps (around the time when subtrial 4 completes enrollment, and around the time when subtrial 3 completes enrollment).

Whatever the scenario, the *Ora* procedure is the one that achieves the expected number of correct success declarations the quickest. As already discussed, the *Ora* procedure is not a practical procedure, because it relies on the unknown true response rates. However, together with the *EFR* procedure, it can serve as a benchmark for the performance of the other allocation procedures.

Figure 2 shows that the procedure based on predictive probabilities ( *PrP* ) is closest to the *Ora* procedure. Like *Ora* , *PrP* allocates each participant t to the prevailing subtrial, which is, in this procedure, the subtrial with the highest predictive probability. Unlike *Ora* , the *PrP* procedure can be applied in practice, because the predictive probability can be obtained from the observed data.

The randomized predictive probability procedures ( *40% -PrP* and *70% -PrP* ) perform more closely to the *EFR* procedure. The larger ε , the closer to *EFR* it gets. This can be consistently observed across all scenarios of Figure 2. We simulated more procedures with other values of ε , but qualitatively the results were the same, so we do not report them here. It may feel counterintuitive that the performance of *ε -PrP* is generally monotone in ε , because we introduced this family of procedures to correct for potential mistake of *PrP* to greedily stick to the prevailing subtrial in unlucky situations, but it seems that introducing randomization cannot improve the expected performance.

#### 4.1.2. Platform trials

In principle, the situation is the same for platform trials, see Figure 3 for the cumulative distribution and Figure 10 in Appendix B for the probability mass. The difference to the umbrella trial is a smaller “frame” (the difference between the curves for the *Ora* and the *EFR* procedures), which depends upon the scenario. This can best be understood by looking at the scenario 1131 (second row of Figure 3).

In a platform trial, the 4 subtrials enter in a staggered manner. In our simulation, each new subtrial enters after 12 periods since the previous one. Since the first two subtrials of this scenario are null ( $\theta_{s}=0.1$ ), the *Ora* procedure will allocate participants 13 to 24 more or less equally between the first two subtrials, and no correct success declarations can be observed. Once subtrial 3 starts enrollment at period 25 , the *Ora* procedure will tend to allocate the next participants to this subtrial, until it completes enrollment. This, however, occurs with a delay of 24 periods (because the first 24 participants were allocated to null subtrials) compared to the same scenario in an umbrella trial, where all subtrials open for enrollment from period 1 .

How the performance of the *EFR* procedure is affected by the platform entry pattern depends on the entry timing of the good subtrials. If good subtrials enter before the null ones, like in scenario 3123, their success declarations are observed much sooner than in umbrella trials. The performance of both the *Ora* and *EFR* however tends to make the frame becomes narrower. Note that this is natural, as there are also fewer opportunities and scope for decision-making, as the number of subtrials concurrently open for enrollment is often smaller in a platform trial than in an umbrella trial with the same total number of subtrials. Beyond this, the qualitative conclusions from Figure 3 are similar to those in Figure 2.

Note that the results would change if we change the entry order of the subtrials. In a scenario like 3111, one would probably see less difference between an umbrella and platform trial. The difference between the two entry patterns becomes more pronounced the later the good subtrial enters the trial: for 3111 the two patterns are very similar, whereas for 1113 the difference is most visible, see Figure 8 in Appendix B.

#### 4.1.3. Perpetual trials

The situation is different for master protocols with perpetual entry pattern, called *perpetual trial*, as shown in Figure 4 with the cumulative distribution and Figure 11 in Appendix B with the probability mass. The total number of correct success declarations within the first 164 periods cannot be predetermined exactly, since more than four subtrials can enter the trial, and the time of entry depends on the results of the previous subtrials. In principle, the *Ora* and the *EFR* procedure determine the frame again, and the performance of the other procedures is somewhere in between, qualitatively ordered as before.

### 4.2. Subtrial duration and enrollment anatomy

The figures in this section present the expected enrollment to subtrials (vertical axis) under each allocation procedure as a function of time (horizontal axis). Each plot of the figure is determined by a response rate vector (rows) and sample size (columns). Within each plot of the figure, there is a row for each subtrial (the top row corresponds to subtrial 4 , the bottom row to subtrial 1 ), and the different colors represent the five allocation procedures. Each expected time described below is always obtained as the average over 50,000 simulations.

The shaded bar represents the expected duration when the corresponding subtrial is in the trial. The bar starts at the expected time of opening for enrollment and ends at the expected time of final analysis. The starting time is predetermined in our simulation results: for umbrella trials, all subtrials are open from period 1, for the platform and perpetual trials, subtrial 1 (bottom) is open from period 1 , subtrial 2 from period 13 , subtrial 3 from period 25 and subtrial 4 from period 37 .

The boxplots exhibit the anatomy of enrollment of participants and their allocation to each subtrial. The left and right whiskers of the boxplots indicate the expected time when the first and the last participant are allocated to the respective subtrial, and the box itself represents the inter-quartile range and the median of the expected time when 25%, 50%, or 75% of the sample size was enrolled to that subtrial.

#### 4.2.1. Umbrella trials

Figure 5 provides deeper insight into how the allocation procedures compare for umbrella trials. Within each scenario (defined by a response rate vector and a sample size), there is no difference in subtrial duration when using the *EFR* procedure (blue). The same is true for all other allocation procedures but in the first row only, where all subtrials have the same response rate $\theta_{s}=0.1$ . However, there are differences in the duration of the subtrials in the other rows, where the four subtrials have different response rates.

**Figure 5.**
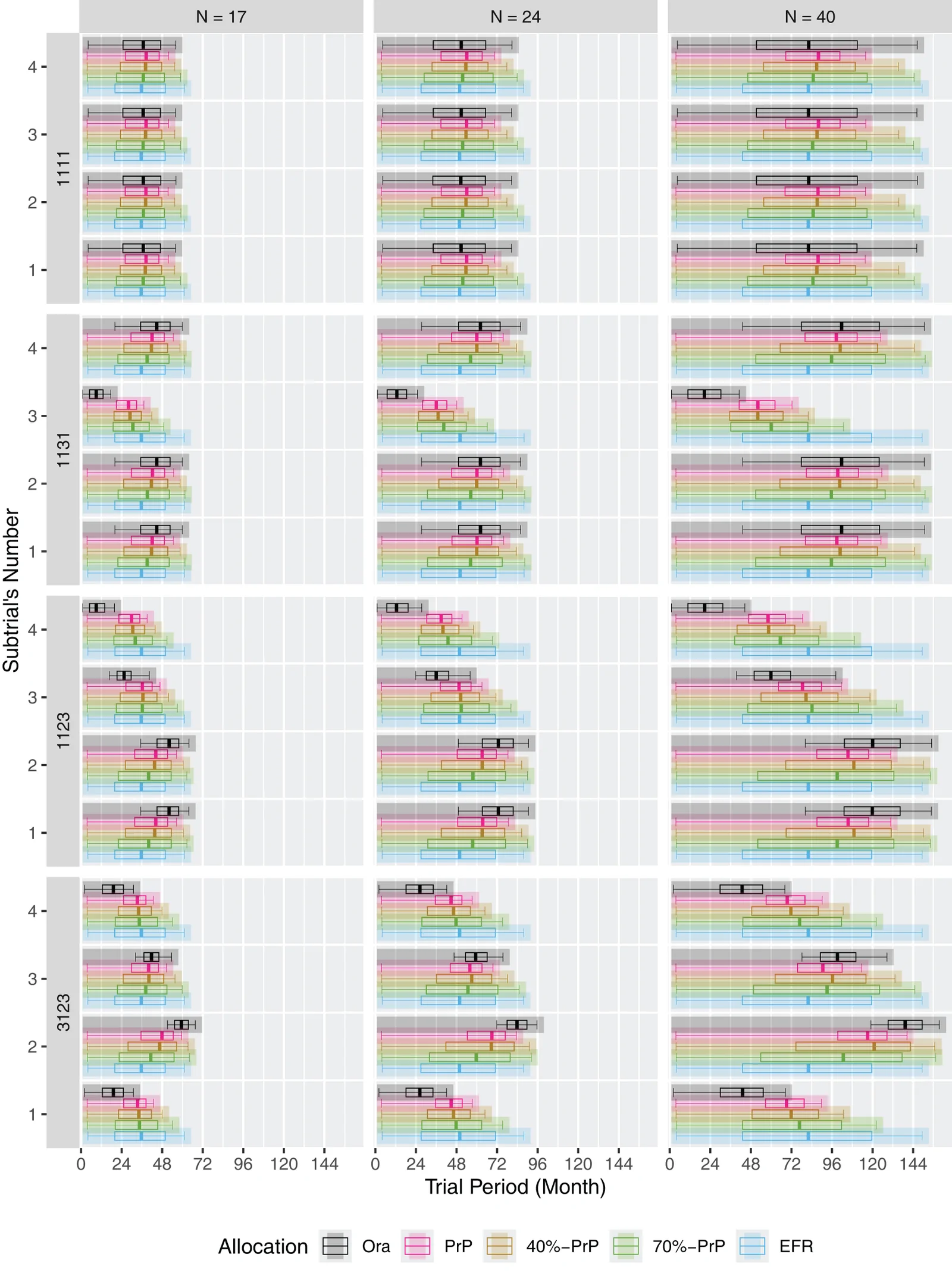
The expected subtrial duration in an umbrella trial.

As an example, take the second row, where subtrial 3 has a response rate $\theta_{3}=0.3$ , while the other three subtrials have a response rate $\theta_{s}=0.1$ . Here, the duration of subtrial 3 is considerably shorter when using the *Ora* procedure (black). The expected duration for the other three subrials is the same under *Ora* and does not differ much from the duration under *EFR* . This behavior of the *Ora* procedure is as intended: by allocating participants to the prevailing subtrial (the subtrial with the highest conditional power), participants are enrolled quicker to the intervention with the highest response rate, because most likely that intervention will be in the prevailing subtrial over time in a scenario like this. Once this subtrial is completed, the procedure behaves more or less like *EFR* . Differences that can occur by chance average out when calculating the expected duration of the subtrials.

Similar findings apply to the third and fourth rows. In the third row, subtrial 4 with the highest response rate $\theta_{4}=0.3$ has the shortest expected duration, subtrial 3 with the second highest response rate $\theta_{3}=0.2$ exhibits the second shortest subtrial duration, and the other two subtrials (with response rates $\theta_{s}=0.1$ ) have an even longer expected duration, which is even longer than the expected duration under *EFR* . In the forth row, there are two best subtrials ( 1 and 4 ). These subtrials have the same expected duration, which is shorter than that of the other subtrials.

In general, the *Ora* procedure achieves what was intended. The expected duration of the best subtrials are shorter than those for the other subtrials. The ordering of the subtrial duration corresponds to the ordering of the response rates. As before, this is of no practical relevance, but it serves as a reference of how efficient allocation could be if one knew the true response rates.

The *PrP* procedure (red) demonstrates behavior similar to the *Ora* procedure. The expected durations of the best subtrials are shorter than those for the other subtrials, and the ordering of the subtrial duration corresponds to the ordering of the response rates. The behavior is just not as pronounced as with the *Ora* procedure.

For example, in the second row, both procedures clearly favor subtrial 3 (with response rate $\theta_{3}=0.3$ ) over the other three subtrials (with response rates $\theta_{s}=0.1$ ). However, the expected duration of subtrial 3 under *Ora* is much shorter than under *PrP* (around 24 versus around 42 when N=17 ). As a consequence, the expected duration of the other three subtrials is shorter under *PrP* as compared to *Ora* .

Table 2 shows that the performance of the *PrP* procedure improves with increasing sample size. The relative time when the last participant is enrolled into the best subtrial gets shorter with increasing sample size. For example in the scenario 1131 with sample size N=17 , the expected period when the last participant is enrolled into subtrial 3 is 37 (with a total number of 68 enrollment periods). One could also say that the expected number of participants enrolled to the umbrella trial by the time the best subtrial completes enrollment is 37 . Since the total number of participants is 68 , this best subtrial completes enrollment when 54.41% of the total number of participants to be enrolled into the trial have been enrolled. The corresponding percentages for an umbrella trial with N=24 or N=40 decrease to 48.96% and 41.88% , respectively. The performance of the *PrP* procedure also improves when the underlying response rates of the best subtrial increase. This can be seen by comparing the performance of the *PrP* procedure between scenarios 1121, 1131, and 1141 within each sample size. The values which the *PrP* procedure achieves for scenario 1141 appear to be pretty remarkable improvements, both in absolute and percentage terms.

**Table 2.** Comparison of the expected time (period, rounded to integer) of the last participant into the best subtrial and the percent of the total sample size needed to enroll this last participant into the best subtrial under the *PrP* procedure in an umbrella trial.

Scenario	N=17	N=24	N=40
1121	47 (69.12%)	59 (61.46%)	98 (61.25%)
1131	37 (54.41%)	47 (48.96%)	67 (41.88%)
1141	32 (47.06%)	42 (43.75%)	57 (35.62%)

Note that the corresponding values for *EFR* ( 89.71% for N=17 , 91.67% for N=24 , and 93.12% for N=40 ) and *Ora* ( 25% across all sample sizes) do not differ across the scenarios. For *EFR* , this is because the value of the response rate in the best subtrial does not matter, and for *Ora* , because the procedure allocates the first N participants to the best subtrial anyway, again regardless of the response rate.

We can also see that the behavior of the randomized *PrP* procedures (brown and green) is qualitatively similar to *PrP* and quantitatively monotone between *PrP* and *EFR* .

#### 4.2.2. Platform trials

The situation is more involved for platform trials with staggered trial entry, as presented in Figure 6. Under *EFR* (blue), the first subtrial to enter will complete first, and the last subtrial to enter will complete last. The expected subtrial duration is longest for the subtrials starting enrollment in the middle of the master protocol trial. This pattern is identical for all response rate vectors.

**Figure 6.**
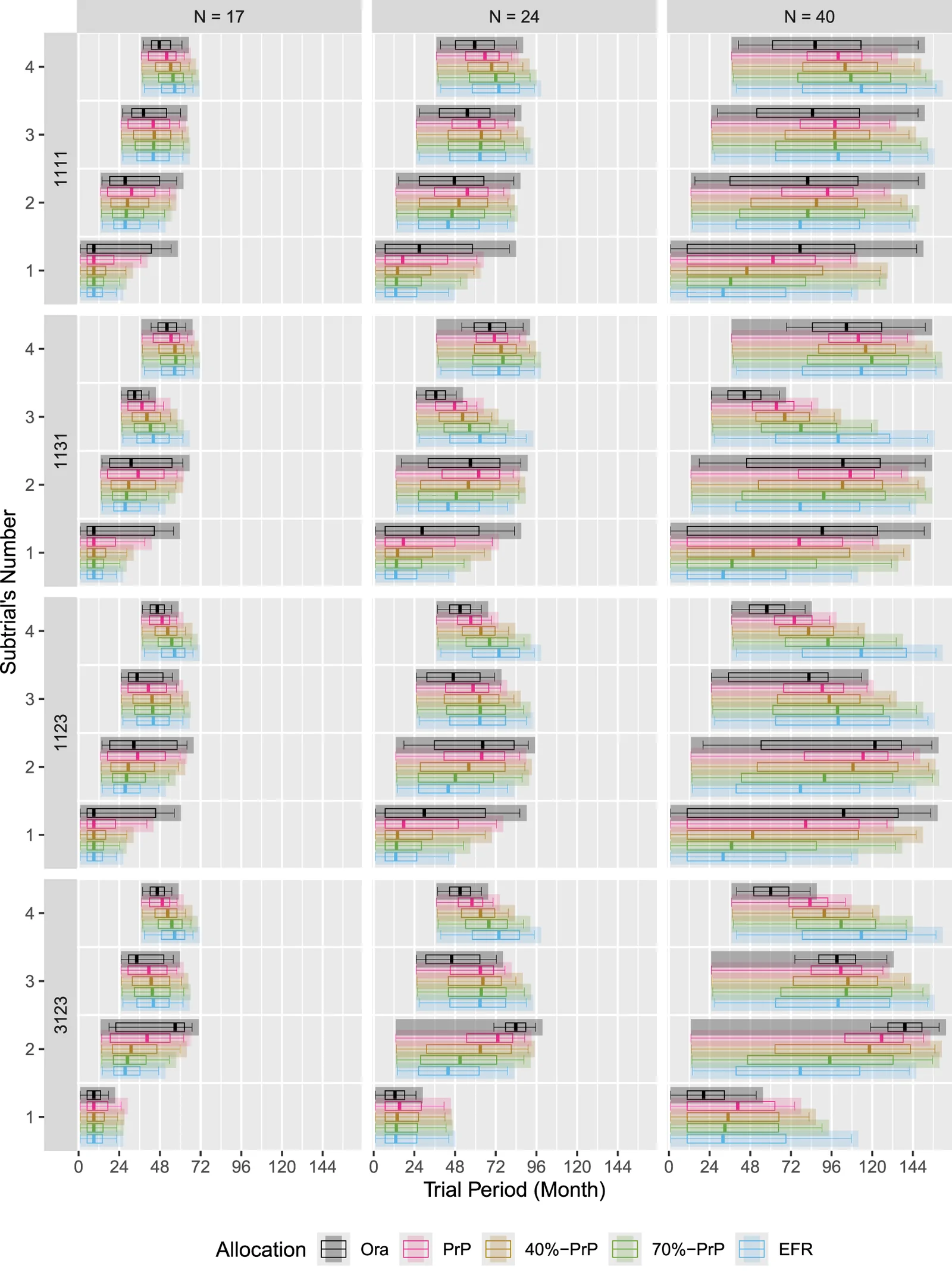
The expected subtrial duration in a platform trial.

In contrast, the *Ora* procedure (black) seems to ensure that subtrials with the same response rate complete around the same time. This can be best seen in the first row, where all subtrials have the same response rate $\theta_{s}=0.1$ . The first subtrial to enter is still the first to complete, and the last to enter is the last to complete, but the difference is much less pronounced. For N=17 , all subtrials complete enrollment on average around period 60 , even subtrial 4 which enter only in period 37 . With larger sample sizes, the expected end of the four subtrials is around period 84 ( N=24 ) and 150 ( N=40 ). A similar pattern can be seen in the second row for subtrials 1 , 2 , and 4 , or in the third row for subtrials 1 and 2 . These subtrials are all null, which is worse than the remaining subtrials, and all these subtrials complete around the same time. In the second row, only the best subtrial ( 3 ) completes much sooner and has a much shorter expected duration, despite being open for enrollment only from period 25 . In the third row, the good subtrials enter the master protocol trial later (subtrial 3 with response rate $\theta_{3}=0.2$ in period 25 , subtrial 4 with $\theta_{4}=0.3$ in period 37 ) than the two null subtrials. They complete around the same time as the latter ones but have considerably shorter expected durations. The benefit of the *Ora* procedure partially depends upon the time when the best subtrial enters the trial, which can be seen in the fourth row with two best subtrials ( 1 and 4 ). These have the same expected duration, but subtrial 1 being the first subtrial to enter has an expected completion before period 24 . Subtrial 4 being the last to enter, has an expected completion before period 55 , which is still sooner than the null subtrial 2 .

One can explain this behavior of the *Ora* procedure by looking at the conditional probability for a subtrial to declare success at the final analysis given the data observed so far. In a situation with four null subtrials like in the first row, the first subtrial will have enrolled 12 participants by the time the second subtrial enters. Since the subtrial is null, there will be many non-responses among these first 12 participants, leading to a small conditional probability to declare success at the final analysis for this subtrial. This in turn will favor the second subtrial in terms of allocation of participants: with fewer observed non-responses, the conditional probability to declare success at the final analysis will tend to be larger for the second subtrial, until it catches up with the number of non-responses. This effect will carry through until the last subtrial enters.

The *Ora* and the *EFR* procedures serve as the frame again. The other procedures demonstrate patterns that are somewhere in between of *Ora* and *EFR* , with *PrP* being the closest to *Ora* , and *70% -PrP* being the closest to *EFR* , as intended.

#### 4.2.3. Perpetual trials

Figure 7 shows the expected subtrial durations for a perpetual trial. Only the first four subtrials to enter are displayed. In principle, we see similar patterns as for platform trials under the different allocation procedures. However, the expected subtrial duration for all the subtrials is considerably longer than that for the subtrials in a platform trial. This is because a subtrial that completes enrollment is replaced in a perpetual trial, increasing the competition about new participants to be allocated. This means that null subtrials tend to enroll slower (as compared to a platform trial) and will remain in the master protocol trial for longer.

**Figure 7.**
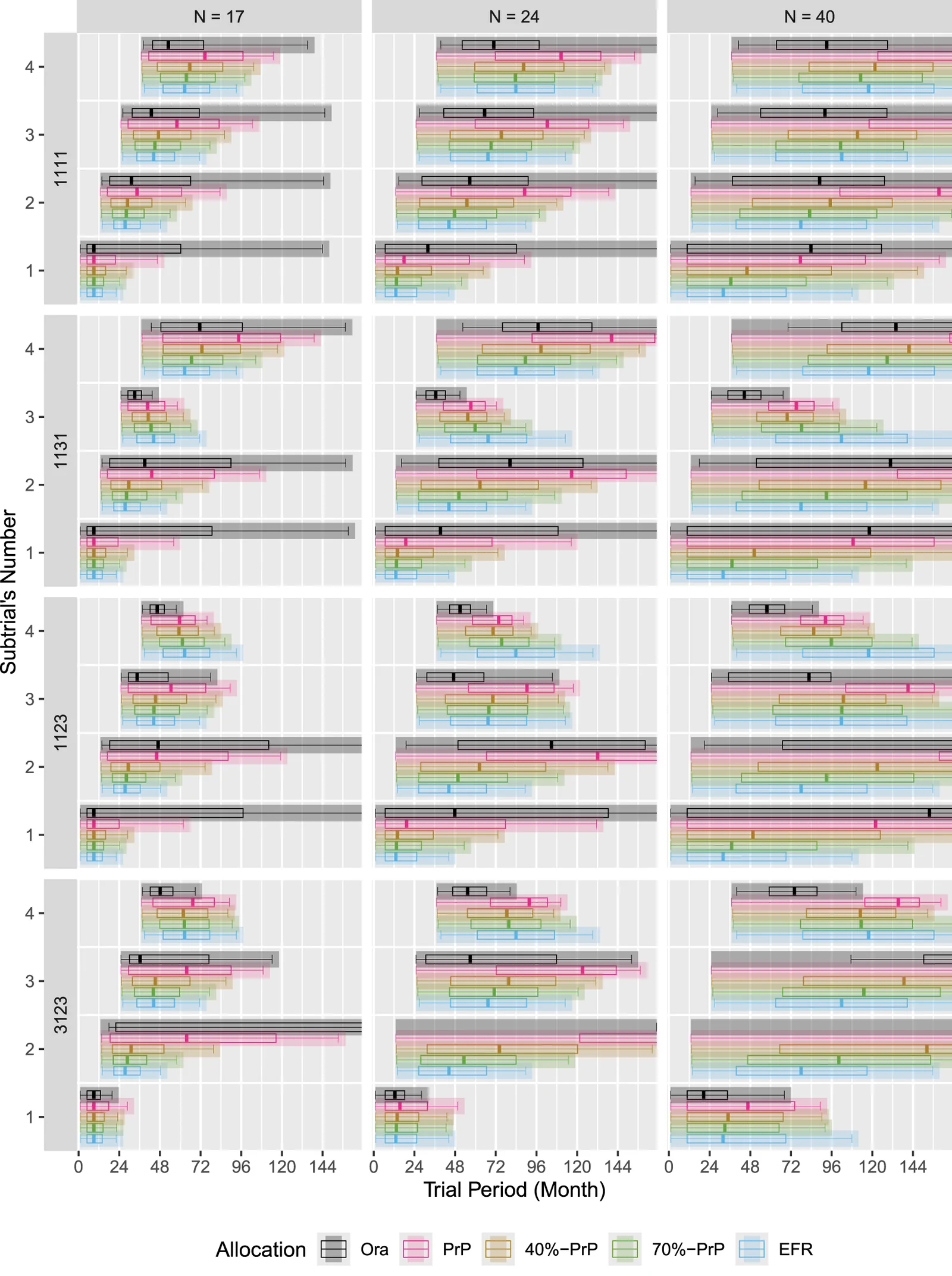
The expected subtrial duration in a perpetual trial.

**Figure 8.**
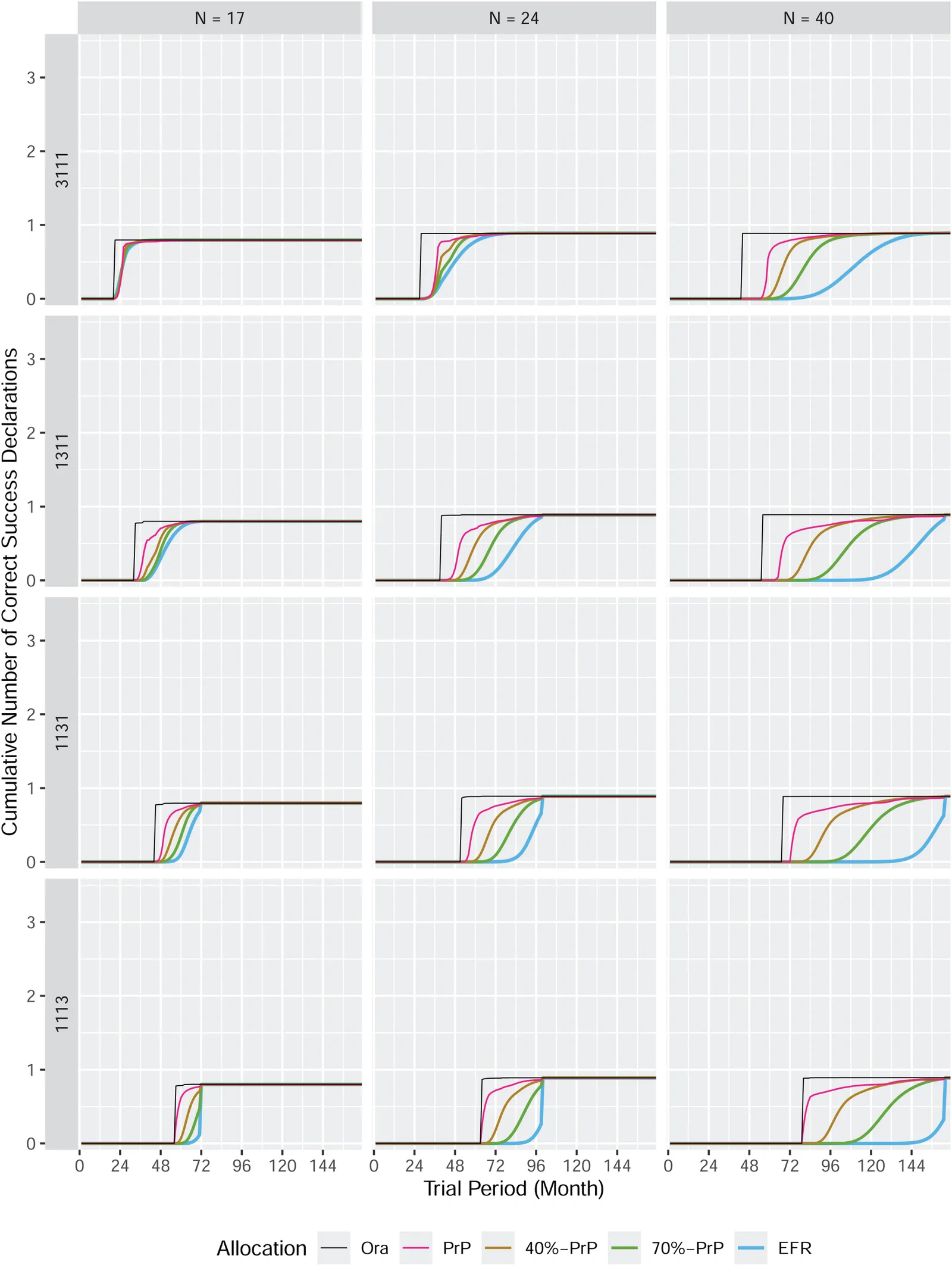
The cumulative number of correct success declarations in a platform trial: variants of scenario 1131.

**Figure 9.**
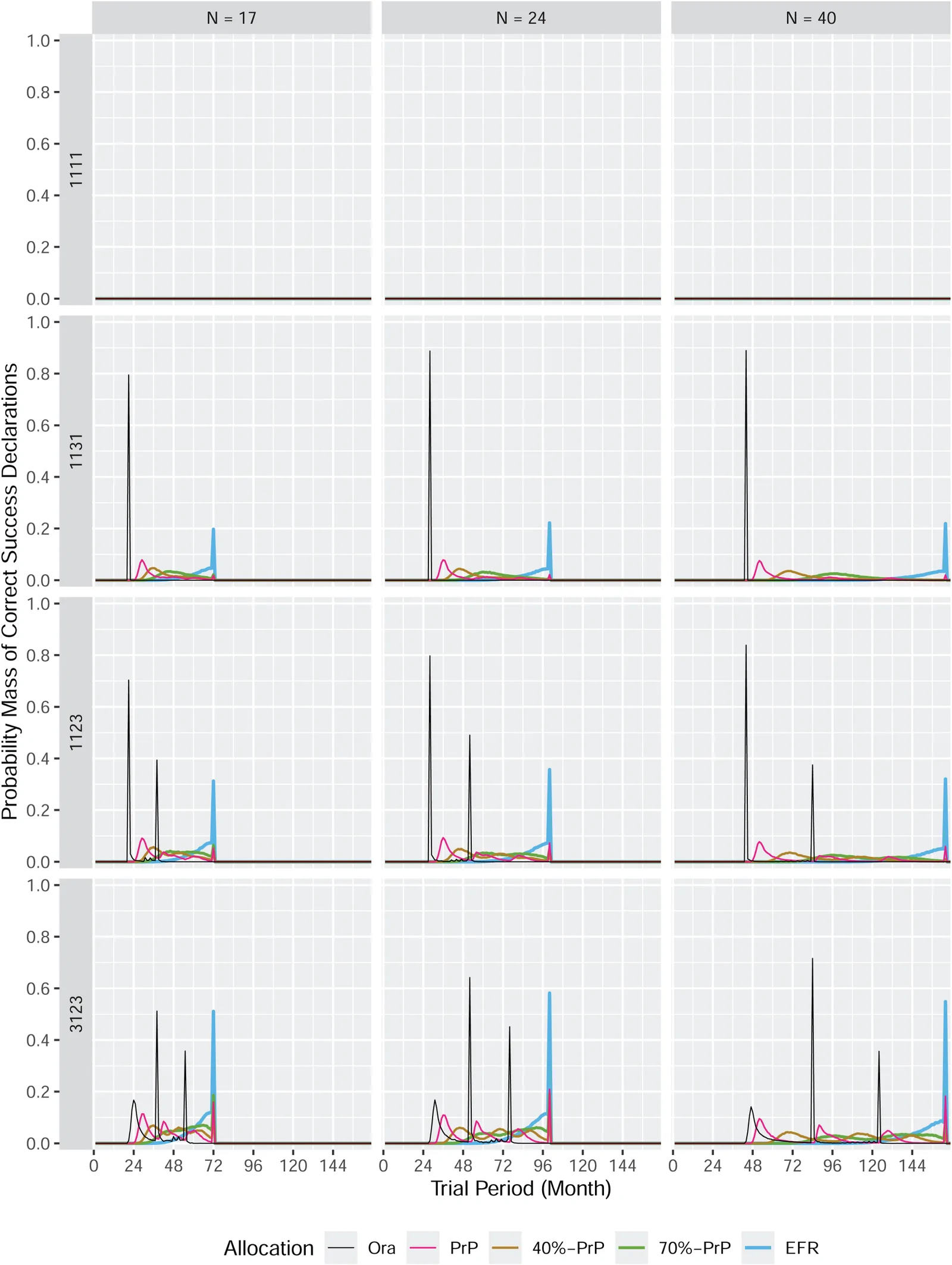
The probability mass of correct success declarations in an umbrella trial.

**Figure 10.**
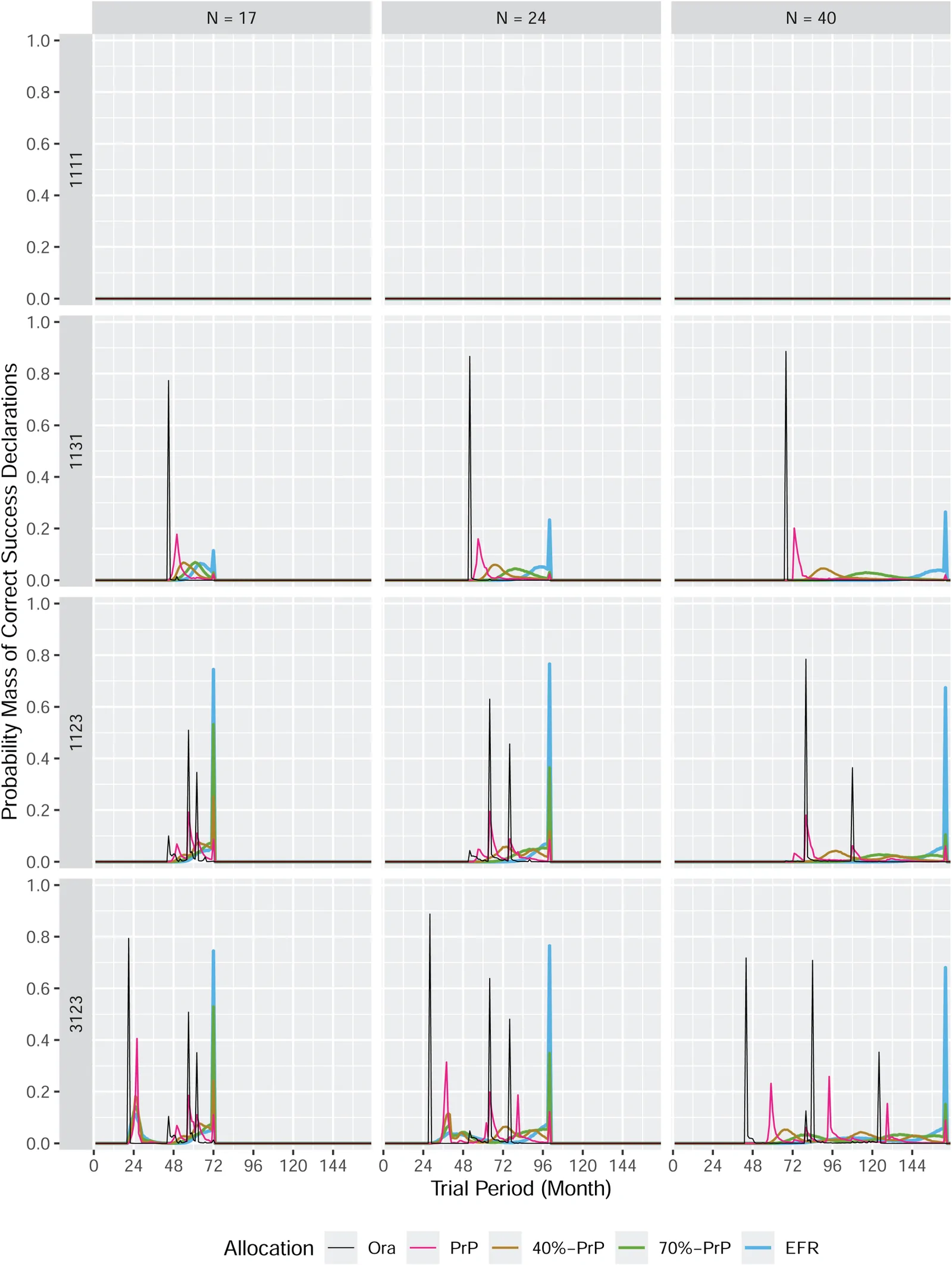
The probability mass of correct success declarations in a platform trial.

**Figure 11.**
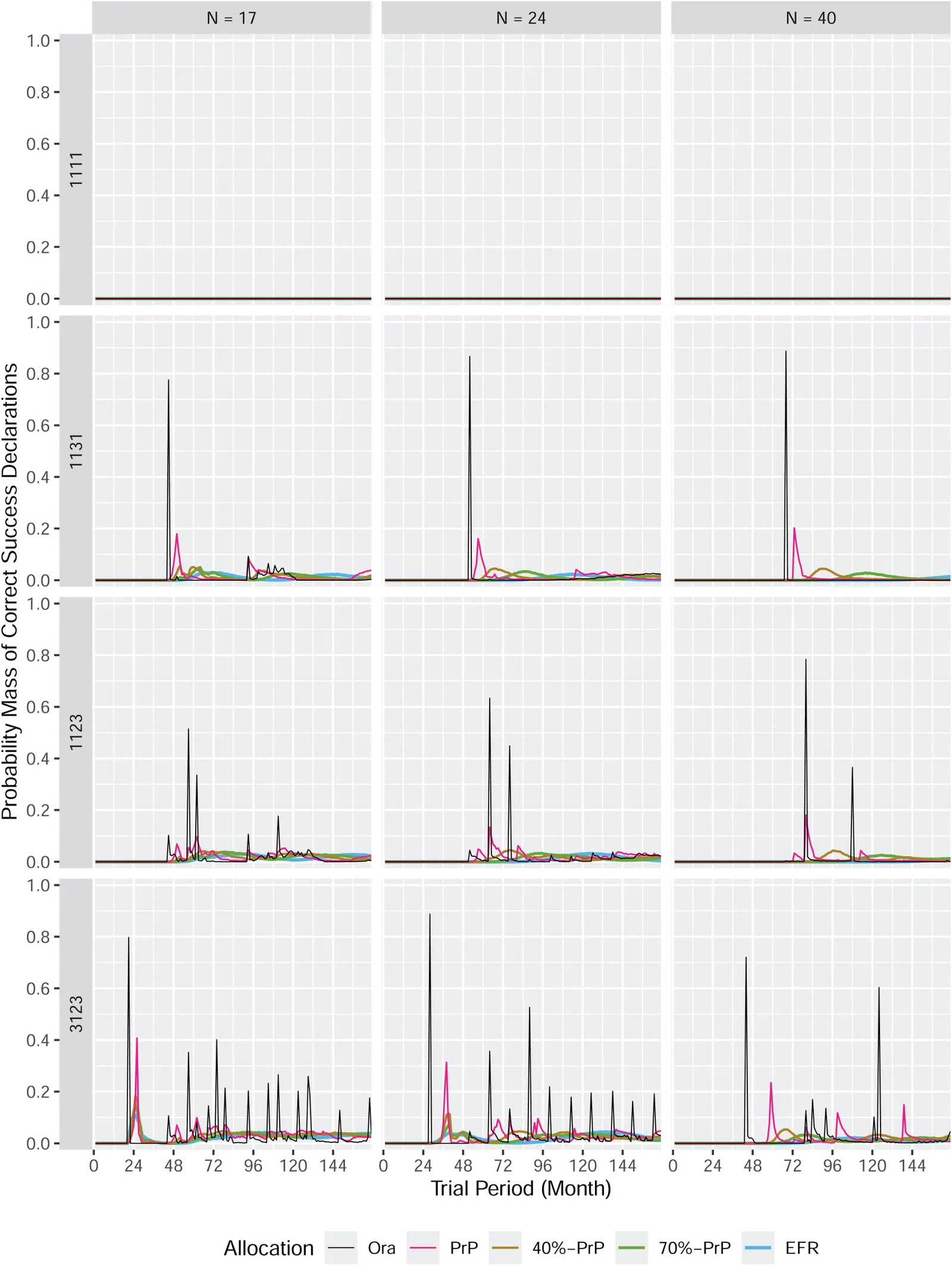
The probability mass of correct success declarations in a perpetual trial.

### 4.3. Additional features of the allocation procedures

In subsections 4.1 and 4.2 we have discussed that the *PrP* procedure is able to complete enrollment to the best subtrial sooner than to the other subtrials. How this works in detail is explained in Section 5 using an umbrella trial and vector of response rates 3123. In this scenario, this feature of the *PrP* procedure is the intended effect. However, by chance this feature may also apply to a null subtrial.

This can be best explained by looking at the number of wrong success declarations as displayed in Figures 12–14 in Appendix B. These figures display similar information as those discussed in subsection 4.1, except that wrong success declarations are presented instead of correct success declarations. A wrong success declaration is a success declaration for a null subtrial (i.e. with response rate $\theta_{s}=0.1$ ).

**Figure 12.**
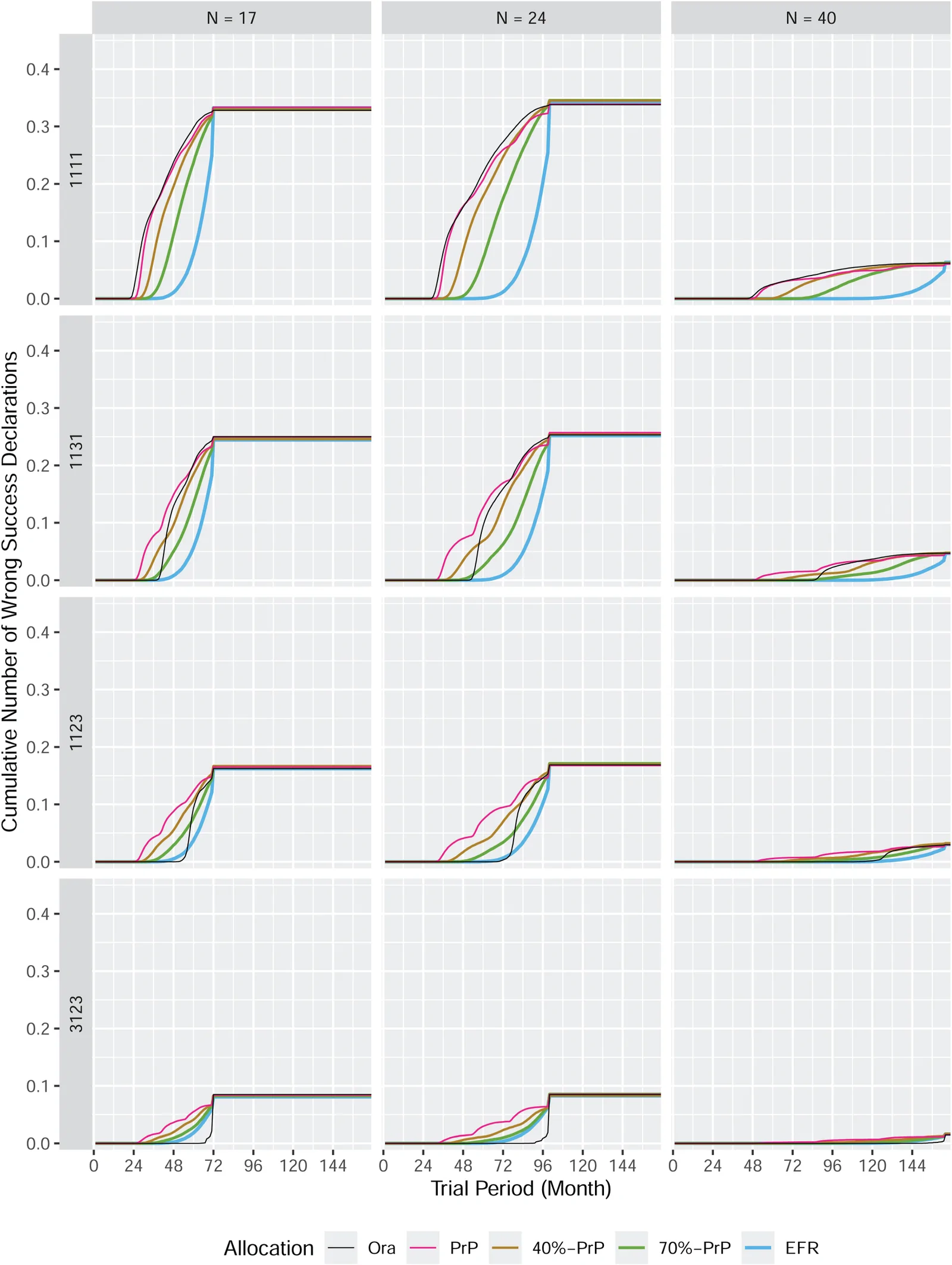
The cumulative number of wrong success declarations in an umbrella trial.

**Figure 13.**
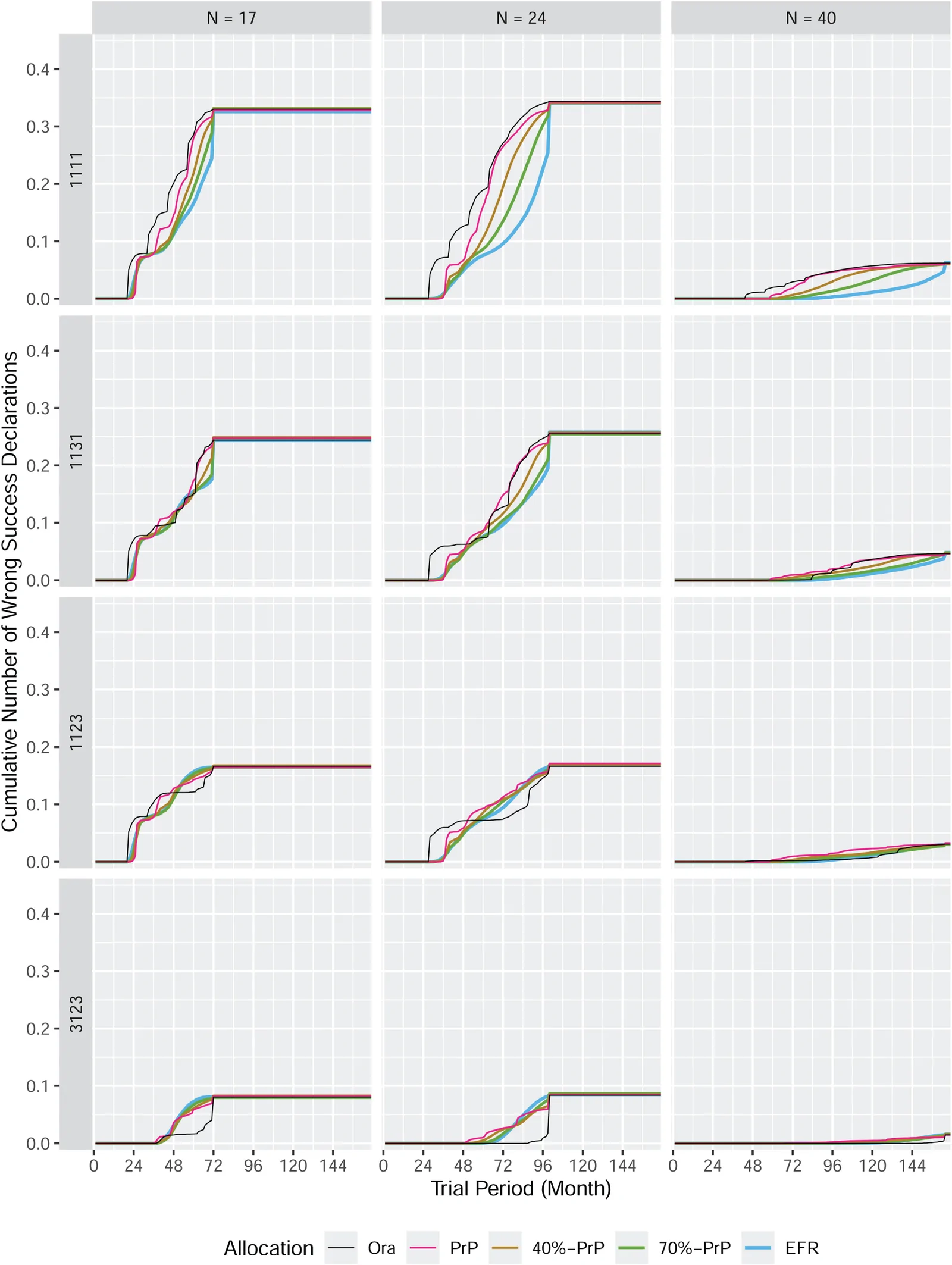
The cumulative number of wrong success declarations in a platform trial.

**Figure 14.**
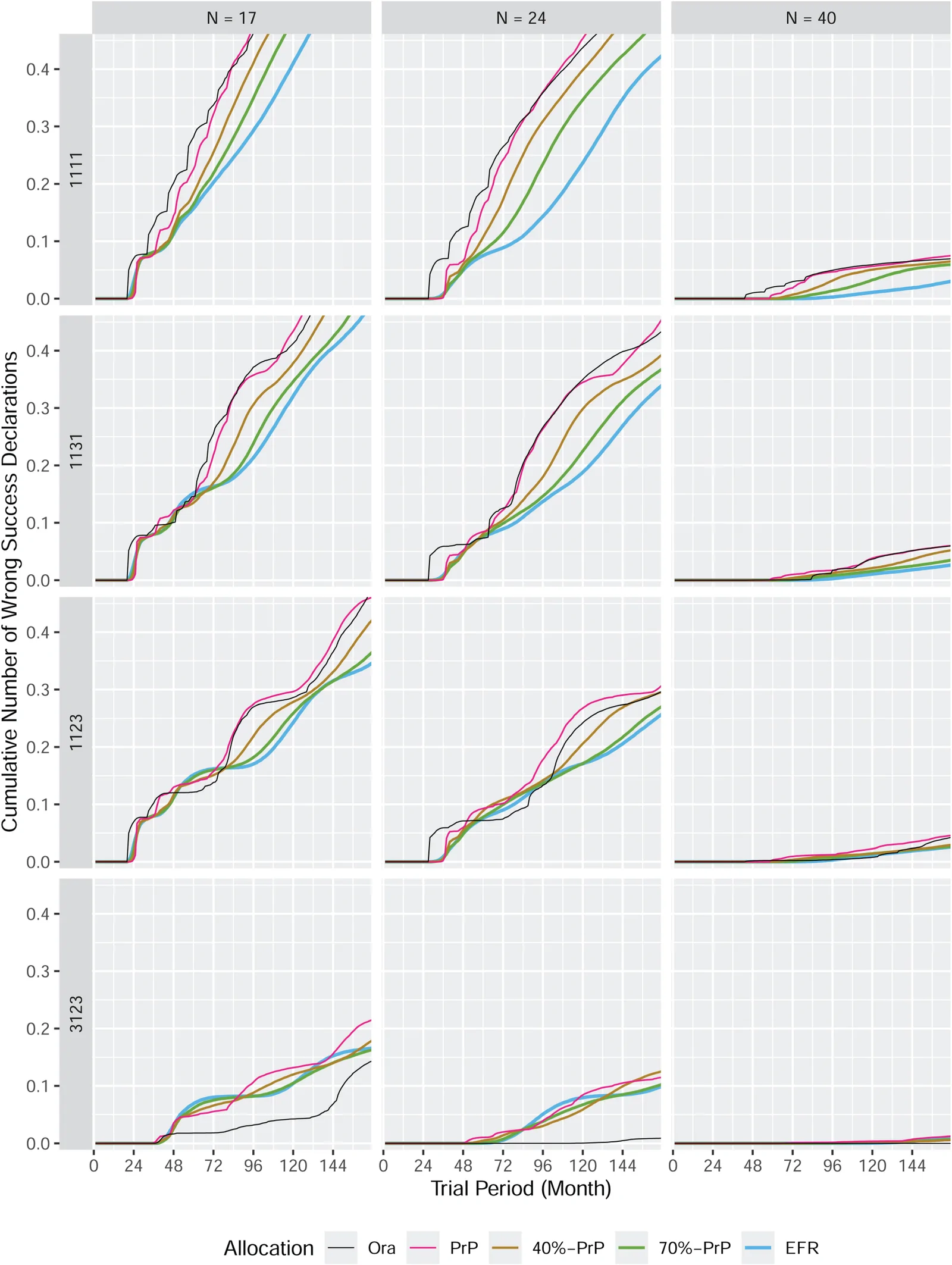
The cumulative number of wrong success declarations in a perpetual trial.

As an example, take the first row of Figure 12 corresponding to the global null scenario 1111. All four subtrials are null, because their true response rate is $\theta_{s}=0.1$ . In this scenario, all success declarations are wrong. The overall expected cumulative number of success declarations can be obtained from Table 1 again, it is four times the number in the first column for this scenario. One can see from the first row of Figure 12 that success declarations under the *EFR* procedure start rather late (around period 42 for N=17 , period 66 for N=24 , and period 126 for N=40 ). Under procedures *Ora* and *PrP* , success declarations start much sooner (around period 24 for N=17 , period 30 for N=24 , and period 48 for N=40 ). This means that these two allocation procedures pick one (the one with highest endpoint observations from the first few allocated participants) subtrial soon despite there is no good subtrial, and complete the enrollment to this subtrial before allocating participants to the other subtrials. This is explained in more detail at the end of Section 5.

One could argue that this is not an issue in the global null scenario 1111, because it does not matter which of the four null subtrials finishes first, or whether they all finish around the same time. However, when looking at the second row of Figure 12 we observe the same behavior of the *PrP* procedure. Here in the 1131 scenario, there is a best subtrial, and a quick declaration of a wrong success means that by chance a subtrial which is not the best subtrial completed enrollment first. This behavior is less pronounced with fewer null subtrials in the scenario, which can be seen by comparing all four rows of this figure.

In a scenario with both good and null subtrials, it is not a desired feature to complete a null subtrial first. This is the motivation to define randomized procedures, and one can see from the three figures that these procedures indeed reduce this undesired feature.

## 5. Example individual simulations

In this section, we present 3 individual simulations that illustrate the behavior of the *PrP* procedure in an umbrella and platform trials, and the behavior of the *Ora* procedure in an umbrella trial. All the examples are for the response rate vector 1123, where subtrials 1 and 2 have response rate $\theta_{s}=0.1$ , subtrial 3 has response rate $\theta_{3}=0.2$ and subtrial 4 has response rate $\theta_{4}=0.3$ , and for the sample size N=24 .

As before, enrollment to the trial is simulated at the rate of 1 new participant per period, and that participant’s endpoint is observed after 4 periods. The simulation of the operation of the trial is that participants are enrolled at the beginning of the period, and the endpoint is observed and analysis is done at the end of the period, setting up the allocation/randomization for the next period. The details are given in Table 3.

### 5.1. Example A: An umbrella trial using **PrP**

In the umbrella trial all subtrials start at the outset. Initially, there is no information on any subtrial so each is assigned a predictive probability of 1, with the result that initial allocation is equal randomization across the 4 subtrials. The first participant’s endpoint is observed during period 5 and included in the update for the start of period 6. Up to this point, the random allocation sequence was subtrial 4, subtrial 4, subtrial 2, subtrial 4, subtrial 1. Randomly, one participant has been allocated to subtrials 1 and 2, and 3 participants to subtrial 4. The observation of the first participant on subtrial 4 is a response and the state of the trial is as follows: subtrials 1, 2 and 3 have predictive probability of success of 1 by virtue of having no observations, and subtrial 4 has a predictive probability of success of 1 as 1 response out of 1 observation has been observed.

So allocation in period 6 is equal randomization across all subtrials, and by chance participant 6 is allocated to subtrial 4. The second participant enrolled (also by chance on subtrial 4) is observed and is a non-response. Subtrials 1 through 3 still have no observations and so are still given a predictive probability of 1, but subtrial 4’s 1 response and 1 non-response gives it a predicted probability of 0.75. Our allocation procedure just allocates to the prevailing subtrial(s), so allocation in period 7 is equal randomization across subtrials 1, 2 and 3.

**Table 3.** Allocations and observations data for example individual simulations of response rates scenario 1123, at the start of period t for each subtrial s : $n_{s}$ is the number of allocated participants, $r_{s}$ is the number of observed responses, $f_{s}$ is the number of observed non-responses, $i_{s}$ is the number of incomplete observations (unavailable due to endpoint delay), $\pi_{s}^{\psi }$ is the probability of success declaration (rounded to 2 decimals, where 0.0+ represents a strictly positive value rounded to 0.00 ) under procedure ψ , and $\lambda_{s}$ is the resulting randomization probability.

	t	6						7						12						17					
Example A	s	$n_{s}$	$r_{s}$	$f_{s}$	$i_{s}$	$\pi_{s}^{PrP}$	$\lambda_{s}$	$n_{s}$	$r_{s}$	$f_{s}$	$i_{s}$	$\pi_{s}^{PrP}$	$\lambda_{s}$	$n_{s}$	$r_{s}$	$f_{s}$	$i_{s}$	$\pi_{s}^{PrP}$	$\lambda_{s}$	$n_{s}$	$r_{s}$	$f_{s}$	$i_{s}$	$\pi_{s}^{PrP}$	$\lambda_{s}$
	1	1	0	0	1	1.00	$\frac{1}{4}$	1	0	0	1	1.00	$\frac{1}{3}$	2	0	1	1	0.00	0	2	0	2	0	0.00	0
	2	1	0	0	1	1.00	$\frac{1}{4}$	1	0	0	1	1.00	$\frac{1}{3}$	1	0	1	0	0.00	0	1	0	1	0	0.00	0
	3	0	0	0	0	1.00	$\frac{1}{4}$	0	0	0	0	1.00	$\frac{1}{3}$	4	0	1	3	0.00	0	4	1	3	0	0.59	0
	4	3	1	0	2	1.00	$\frac{1}{4}$	4	1	1	2	0.75	0	5	1	3	1	0.59	1	9	2	3	4	0.86	1

	t	1						45						61						71					
Example B	s	$n_{s}$	$r_{s}$	$f_{s}$	$i_{s}$	$\pi_{s}^{Ora}$	$\lambda_{s}$	$n_{s}$	$r_{s}$	$f_{s}$	$i_{s}$	$\pi_{s}^{Ora}$	$\lambda_{s}$	$n_{s}$	$r_{s}$	$f_{s}$	$i_{s}$	$\pi_{s}^{Ora}$	$\lambda_{s}$	$n_{s}$	$r_{s}$	$f_{s}$	$i_{s}$	$\pi_{s}^{Ora}$	$\lambda_{s}$
	1	0	0	0	0	0.09	0	0	0	0	0	0.09	$\frac{1}{2}$	8	0	8	0	0.02	0	8	0	8	0	0.02	1
	2	0	0	0	0	0.09	0	0	0	0	0	0.09	$\frac{1}{2}$	8	1	3	4	0.13	1	18	1	13	4	0.0+	0
	3	0	0	0	0	0.54	0	20	1	15	4	0.01	0	20	1	19	0	0.0+	0	20	1	19	0	0.0+	0
	4	0	0	0	0	0.89	1	24	9	15	0	NA	NA	24	9	15	0	NA	NA	24	9	15	0	NA	NA

	t	25						30						37						41					
Example C	s	$n_{s}$	$r_{s}$	$f_{s}$	$i_{s}$	$\pi_{s}^{PrP}$	$\lambda_{s}$	$n_{s}$	$r_{s}$	$f_{s}$	$i_{s}$	$\pi_{s}^{PrP}$	$\lambda_{s}$	$n_{s}$	$r_{s}$	$f_{s}$	$i_{s}$	$\pi_{s}^{PrP}$	$\lambda_{s}$	$n_{s}$	$r_{s}$	$f_{s}$	$i_{s}$	$\pi_{s}^{PrP}$	$\lambda_{s}$
	1	13	2	11	0	0.27	0	13	2	11	0	0.27	0	13	2	11	0	0.27	0	13	2	11	0	0.27	0
	2	11	2	5	4	0.73	0	11	3	8	0	0.79	1	14	3	11	0	0.60	0	14	3	11	0	0.60	0
	3	0	0	0	0	1.00	1	5	0	1	4	0.00	0	9	2	3	4	0.86	0	9	2	7	0	0.57	1
	4	–	–	–	–	NA	NA	–	–	–	–	NA	NA	0	0	0	0	1.00	1	5	0	1	4	0.00	0

Over the next 5 periods, non-responses come in for every subtrial and eventually, at the start of period 12 every subtrial has at least one observation. The first participants on subtrials 1, 2 and 3 result in non-responses, and so allocation becomes restricted to subtrial 4 with its 1 response and 3 non-responses. In period 16 the last incomplete participant on subtrial 3 is observed and is a response. For one period there is equal randomization between subtrial 3 and subtrial 4, both having 1 response and 3 non-responses. The participant arriving in period 17 is allocated to subtrial 4 and period 12’s participant is observed and adds a response to subtrial 4.

From this point on, allocation is solely to subtrial 4 until it is fully enrolled in period 31. It already has 3 responses, while 5 responses are sufficient to guarantee success at final analysis and in this instance that occurs in period 22 (suggesting that early subtrial stopping for success could be useful in this setting). From then on, it has a predictive probability of 1.

Notice how the *PrP* procedure is quite extreme, as it is very sensitive to initial data and once there is a subtrial more likely to declare success than others, it will allocate to it to the exclusion of all others until its enrollment is complete unless the delayed endpoint observations on the other subtrials happen to be high-valued. This often gets good subtrials done quickly, but at the risk of a good subtrial having to wait its turn just because its initial observations are low.

### 5.2. Example B: An umbrella trial using **Ora**

The umbrella trial using the *Ora* procedure is from the same scenario as the previous example. We see the difference in the allocation in period 1, because *Ora* is allowed to know the true response rate that is being simulated. It can calculate an unconditional probability from the outset, hence subtrials with no data are not assigned a probability of 1, a conditional power is calculated based on the true response rate.

Hence subtrial 4 is allocated to exclusively from the outset. Enrollment to subtrial 4 completes in period 24, with the first 24 participants in the trial allocated to it. What happens next is not so straightforward though. Initially of course subtrial 3 is allocated to, as the subtrial with the next highest response rate. However only one response is observed in the first 16 participants on subtrial 3, and in period 45, even knowing that the true response rate for the remaining 8 participants on subtrial 3 (4 of whom have been allocated to subtrial 3 already but have not been observed) is $\theta_{3}=0.2$ , the other subtrials with a true response rate of only $\theta_{s}=0.1$ have a greater conditional probability.

Subtrials 1 and 2 are now allocated to. As the observations of the participants already allocated to subtrial 3 arrive, they are all non-responses. Not surprisingly, since the simulated response rate is only 0.1, the observations for participants on subtrials 1 and 2 are non-responses, until period 61 when a response is observed on subtrial 2.

All allocation is now to subtrial 2 until the number of non-responses observed, and the shrinking number of possible future observations means that, at the start of period 71, subtrial 1 gains the mantle of highest predictive probability, despite having no responses.

Allocation is then to subtrial 1 for the next 10 periods until at period 81 it is shown that it too has a lower conditional probability than subtrial 3. Allocation then switches back to subtrial 3, which completes to 24 enrolled, with one more response, but not enough to make the subtrial to declare success. The trial completes allocating to subtrials 1 and 2, all of which are non-responses.

### 5.3. Example C: A platform trial using **PrP**

The last individual simulation we present is in the same response scenario (1123), using *PrP* in the platform trial setting, with the 4 subtrials arriving over time.

In this setting, at the outset only subtrial 1 is present, so all allocation is to subtrial 1 until subtrial 2 joins the trial at the start of period 13. Until it has observation data, subtrial 2 is given a predictive probability of 1, and so is allocated the next 4 participants. In period 17 the first participant on subtrial 2 is observed and it is a non-response, meanwhile there has been observed 2 responses and 10 non-responses on subtrial 1. Allocation switches to subtrial 1 for 1 period, but the second observation on subtrial 2 is a response and allocation remains with subtrial 2 until period 25 when subtrial 3 enters.

From period 25 allocation is to subtrial 3 as it has no observations data and is given a predictive probability of 1. In period 30, the first observation on subtrial 3 becomes available and is a non-response, and allocation returns to subtrial 2. By the start of period 33, 4 of the first participants on subtrial 3 have been observed with 2 responses and 2 non-responses, and allocation switches back to subtrial 3 until period 37 when subtrial 4 enters.

Subtrial 4 starts with no observations data and given a predictive probability of 1. In period 41 the first observation on subtrial 4 becomes available and it is a non-response, so allocation switches back to subtrial 3. However, once the first 4 participants on subtrial 4 are observed, there are 2 responses and 2 non-responses, and allocation returns to subtrial 4 where it remains until subtrial 4 completes enrollment. Allocation then returns to subtrial 3 until it too completes enrollment. Finally, allocation switches back and forth between subtrial 1 and 2 until both complete (and fail to declare success).

### 5.4. Discussion

These accounts of individual simulations hopefully make clear how the allocation procedures behave. Where trials use designs with some complexity, analyzing individual simulations is crucial to understanding how the adaptations perform in practice (and not just the average), for debugging the simulation code (looking at individual simulations exposed two bugs in our implementation, well after we were happy with the overall operating characteristics of the simulations), and for conveying the design to others.

A feature of the *PrP* procedure that we did not explore was how long and how favorable to make the “run-in”. This is the phase of prioritized allocation to a new subtrial when it enters the trial. We chose to give it a predictive probability of 1 until it had observations data, which in our setting was after 5 periods. If no other subtrial has predictive probability of 1, the new subtrial gets 5 participants allocated to it; otherwise it competes with them using equal randomization. Options would have been to give it some other predictive probability: greater than 1 to give it absolute priority, 0.75 or 0.5 , say, to give it lower priority, and to explore other lengths of run-in.

## 6. Conclusion

In this paper we have introduced participant allocation and randomization procedures for master protocols that consist of single-arm subtrials. The objective of these procedures is to allocate participants sooner to those subtrials that are more likely to declare success. We have simulated the performance of these procedures under different patterns of subtrial entry to the master protocol, for different sample sizes, and for different scenarios of underlying response rates, and compared their performance to that of two reference procedures: equal fixed randomization across subtrials (which is a realistic alternative for such master protocols), and an allocation procedure that is based on knowledge of the true underlying response rates of the subtrials (which is not implementable in real life but serves as a reference for how soon the subtrials that are most likely to declare success could complete enrollment). To perform the simulations, we have developed an open-source clinical trial designer and simulator “Single-arm.”^21^

Our simulations show that the procedure that allocates each participant to the subtrial with the highest predictive probability of current study success is able to considerably speed-up enrollment to subtrials that are most likely to declare success. The relative performance of this procedure improves with increasing sample sizes per subtrial, and with increasing response rates in the subtrial with the intervention with highest response rate, ceteris paribus. It outperforms the randomized procedures which were tested in our simulation study. The *PrP* procedure may be very valuable for master protocols with rare diseases, where there is a high medical need and patients are sparse. In such situations it is crucial to get efficacious interventions to the market as soon as possible, and to use the available participants as effectively as possible to develop such interventions.

The randomized procedures that were tested are mixtures between the predictive probability procedure and equal randomization. They are not quite as efficient as the *PrP* procedure in quickly allocating participants to the subtrial(s) that are most likely to declare success, but they include an element of randomization, which may be important in some clinical trials.

Our simulation study was set up for small sample sizes, which was driven by one of our motivating examples in a rare disease with a binary endpoint. In such a situation, the power does not increase monotonically with increasing sample sizes when fixing the underlying response rate, see Chernick and Liu.^30^ The benefit of the procedure would be even more pronounced when looking at larger sample sizes, or when considering situations with a continuous endpoint. In any case, for binary endpoints one needs to carefully consider which sample size to use.

The procedures considered in our simulation study are based on the predictive probability to declare success in the final analysis given the available data when a new participant is to be randomized. Ultimately, it does not matter how this predictive probability is being calculated, whether according to a frequentist approach (as in this paper), or using a Bayesian approach.^31^ The type of endpoint does not matter either, and by using a neutral scale like predictive probabilities one can even combine subtrials with different types of endpoints when applying the procedure. The event of the predictive probability (current study success) may also be adjusted to other objectives of the master protocol trial, for instance, identifying the most efficacious intervention or an intervention with the highest predicted probability of success of a future trial, the whole development program, adoption by the out-of-trial patient population, etc.

The family of allocation procedures offers a potential for further investigation and improvements when considering additional metrics. For instance, ideas for allocation procedures from the literature on the multi-armed bandit problem^32^ might be worth exploring and may potentially be tweaked to fit this particular setting. Furthermore, we have taken a very naïve approach to tie-breaking in *PrP* or to randomizing in *ε -PrP* using *EFR* . More sophisticated tie-breaking and randomizing approaches may improve the performance of these allocation procedures.

There are several directions in which the proposed allocation procedures or the setting of the simulation study could be extended, but we have kept the paper streamlined with the aim of focusing on the foundational methodological ideas. There is therefore room for introducing such extensions either in future research or when employing these in a particular disease context, which would typically be motivated by practical considerations for specific disease, and not considered all at the same time. Such features include: allowing early subtrial stopping on the basis of success declaration once the required number of responses is observed; allowing early subtrial stopping on the basis of futility (both of which could help to further accelerate screening for efficacious interventions and might mitigate some of the concerns against response-adaptive allocation); allowing early endpoint observations in a schedule of visits; employing other randomization schemes than the complete randomization we have used in order to avoid extreme imbalance in participants’ allocations; development of distributed, multi-center implementation of the proposed allocation procedures as master protocol trials for rare diseases will likely be implemented as multi-center trials.

## Data Availability

The datasets generated and analyzed during the current study are available from the corresponding author on reasonable request.

## Appendix A: Methodology details

In this section we briefly describe how the predictive probability of success is obtained for our simulation study. The approach is based on the EU-PEARL trial and the methodology described in Heimann et al.,^20^ which we briefly present here to make this paper self-contained.

### A.1 Final analysis based on confidence distributions

Following Section 3.1 of Heimann et al.^20^, we first describe how the final analysis is assumed to be performed, using a method based on confidence distributions. We consider a single-arm subtrial s with prespecified sample size $N_{s}$ . Let $t^{*}$ be the time point when the final analysis is performed, which, abusing notation, corresponds to the moment just before the enrollment of participant $t^{*}$ such that the number of observations reaches the sample size for the first time, that is $n_{s}(t^{*})=N_{s}$ while $n_{s}(t^{*}-1)<N_{s}$ . Let us denote the observed endpoint cumulative sum used in the final analysis by $Y_{s}^{*}=Y_{s}(t^{*})$ . In the rest of the section we focus on one subtrial and drop the subtrial index s .

The final analysis of a single-arm study is based on a *confidence distribution*

(5)
$$H_{N}(p|Y^{*}):=\Phi \left(\frac{p-{\hat{p}}_{N}(Y^{*})}{{\hat{\sigma }}_{N}(Y^{*})}\right)\text{ for }p\in R,$$

which is a normal distribution with mean equal to the observed mean ${\hat{p}}_{N}(Y^{*})=Y^{*}/N$ and standard deviation ${\hat{\sigma }}_{N}(Y^{*})$ which is equal to the standard error of the mean. In the above formula, Φ is the distribution function of a standard normal distribution. Confidence distributions are always data driven distributions on the parameter space. For an introduction to this frequentist concept, we refer to Xie et al.^33^

For a binary endpoint, like in our simulation study, $H_{N}(p|Y^{*})$ defines a distribution on the parameter space p∈[0,1] asymptotically. With this, one can now define a decision rule for the final analysis as

(6)
$$H_{N}(p_{0}|Y^{*})<\alpha \;\text{ and }\;H_{N}(p_{1}|Y^{*})<\beta .$$

The first condition is equivalent to a standard hypothesis test for $H_{0}:p\leq p_{0}$ , with α∈[0,1] being the one-sided significance level. We call $p_{0}$ the *ineffective response rate*. The second condition can be interpreted as that the observed response rate needs to exceed some value near the *desired response rate* $p_{1}$ defined in a data-dependent way by β∈[0,1] . In the special case when β=0.5 , the second condition is identical to ${\hat{p}}_{N}(Y^{*})>p_{1}$ ; when β<0.5 , ${\hat{p}}_{N}(Y^{*})$ will need to be larger. We can assume $p_{1}\geq p_{0}$ without loss of generality (otherwise they can be swapped). As a consequence, if β≤α , satisfaction of the second condition implies satisfaction of the first condition, so we can assume β≥α without loss of generality.

It can be shown that the decision rule (6) is equivalent to $Y^{*}\geq c_{N}$ for an appropriately chosen critical value $c_{N}$ . Many other analysis methods will result in a decision rule with such a monotonicity property, which notably simplifies calculations of the operating characteristics (see Section 4 of Heimann et al.^20^) as well as of the quantities needed for allocation procedures (see below).

### A.2 Calculation of frequentist predictive probability

Following Section 3.2 of Heimann et al.^20^, we now describe the calculation of the frequentist predictive probability. A predictive distribution given the endpoint observations data of n<N participants at an interim time point t (at which n(t)=n and the cumulative number of responses Y(t)=Y ) is a distribution on the integers z∈{0,…,N−n} , the possible number of responses in the remaining N−n observations. A frequentist predictive distribution is a mixture of binomial distributions with probability mass

(7)
$$g^{PrP}(z|N,n,Y)=\sum_{y=0}^{n}\;b\left(y|n,{\hat{p}}_{n}(Y)\right)\cdot b(z|N-n,{\hat{p}}_{n}(y)),$$

where $b(z|N-n,{\hat{p}}_{n}(y))$ denotes the binomial probability to observe exactly z responses in the remaining N−n observations given an estimated response rate ${\hat{p}}_{n}(y)=y/n$ for each theoretically possible number of responses y in the first n observations. Correspondingly, the mixing weight $b(y|n,{\hat{p}}_{n}(Y))$ corresponds to the binomial probability to observe exactly y responses in the first n observations, given the observed response rate ${\hat{p}}_{n}(Y)$ .

Assuming that the decision rule is monotonous and can be expressed as $Y^{*}\geq c_{N}$ (like the one based on confidence distributions above), one can express the predictive probability of success after N participants given the data of n<N participants as

(8)
$$1-G^{PrP}(c_{N}-Y-1|N,n,Y),$$

where $G^{PrP}(\cdot )$ is the cumulative distribution function of the probability mass $g^{PrP}(\cdot )$ .

### A.3 Calculation of conditional probability

With the notation from above, the calculation of the conditional probability for the Oracle procedure is based on the probability mass

(9)
$$g^{Ora}(z|N,n,\theta )=b(z|N-n,\theta ),$$

and therefore the conditional probability for a monotonous decision rule is

(10)
$$1-G^{Ora}(c_{N}-Y-1|N,n,\theta ).$$

## Appendix B: Additional simulation results

In this section we present additional simulation results discussed in Section 4.
